# Mutagenic Effect of Proton Beams Characterized by Phenotypic Analysis and Whole Genome Sequencing in Arabidopsis

**DOI:** 10.3389/fpls.2021.752108

**Published:** 2021-10-28

**Authors:** Sang Woo Lee, Yu-Jeong Kwon, Inwoo Baek, Hong-Il Choi, Joon-Woo Ahn, Jin-Baek Kim, Si-Yong Kang, Sang Hoon Kim, Yeong Deuk Jo

**Affiliations:** ^1^Advanced Radiation Technology Institute, Korea Atomic Energy Research Institute, Jeongeup-si, South Korea; ^2^Department of Plant Science and Technology, Chung-Ang University, Anseong, South Korea; ^3^Department of Horticulture, Chonbuk National University, Jeonju-si, South Korea; ^4^Department of Horticulture, College of Industrial Sciences, Kongju National University, Yesan-gun, South Korea

**Keywords:** proton beams, mutation, Arabidopsis, irradiation dose, DNA structural variation, inversion, gamma-rays, non-homologous end joining

## Abstract

Protons may have contributed to the evolution of plants as a major component of cosmic-rays and also have been used for mutagenesis in plants. Although the mutagenic effect of protons has been well-characterized in animals, no comprehensive phenotypic and genomic analyses has been reported in plants. Here, we investigated the phenotypes and whole genome sequences of Arabidopsis M_2_ lines derived by irradiation with proton beams and gamma-rays, to determine unique characteristics of proton beams in mutagenesis. We found that mutation frequency was dependent on the irradiation doses of both proton beams and gamma-rays. On the basis of the relationship between survival and mutation rates, we hypothesized that there may be a mutation rate threshold for survived individuals after irradiation. There were no significant differences between the total mutation rates in groups derived using proton beam or gamma-ray irradiation at doses that had similar impacts on survival rate. However, proton beam irradiation resulted in a broader mutant phenotype spectrum than gamma-ray irradiation, and proton beams generated more DNA structural variations (SVs) than gamma-rays. The most frequent SV was inversion. Most of the inversion junctions contained sequences with microhomology and were associated with the deletion of only a few nucleotides, which implies that preferential use of microhomology in non-homologous end joining was likely to be responsible for the SVs. These results show that protons, as particles with low linear energy transfer (LET), have unique characteristics in mutagenesis that partially overlap with those of low-LET gamma-rays and high-LET heavy ions in different respects.

## Introduction

Life on Earth has been exposed to ionizing radiation during its evolutionary history (Todd, 1994). Ionizing radiation, along with UV radiation, is believed to have stimulated selective and/or adaptive evolution of organisms by inducing damage or mutations in the DNA (Karam et al., 2001). The background ionizing radiation includes radiation from cosmic and geologic sources (Caplin and Willey, 2018). Cosmic radiation from the sun and extra-solar sources consists primarily of protons (87%) and helium (11%) (Eisenbud and Gesell, 1997). The cosmic radiation dose rates that reach the Earth’s surface fluctuate because of events such as enhanced solar activity or reversion of the Earth’s geomagnetic polarity (Atri and Melott, 2014). It has been postulated that periodic increases in cosmic radiation dose rates may be related to the approximately 62-million-year cycles over which global biodiversity increases and decreases (Rohde and Muller, 2005). Radiation from geologic sources also may have contributed to the evolution of life, especially early life forms. When eukaryotes first emerged, the levels of beta and gamma radiation from geological sources were estimated to be five times higher than the present levels (Gensel, 2008). Analyses of the mutagenic effects of radiation may contribute to an in-depth understanding of the evolution that has led to the diversification of plants and the optimization of biological mechanisms to cope with the biological effects of radiation.

Besides the importance of radiation in the evolutionary process, the characteristics and mechanism of radiation-induced mutations in plants are of great interest because irradiation technology has been widely applied in agriculture and functional genomics studies (Tanaka et al., 2010). Mutation breeding, in which irradiation mutagenesis followed by selection is performed to obtain cultivars with improved characteristic(s), is one of the primary applications of irradiation technology in agriculture (Shu et al., 2012). Traditionally, gamma-rays and X-rays, which are electromagnetic radiations, have been used for mutagenesis during the long history (>90 years) of plant mutation breeding. More recently, ionizing particles, such as fast neutrons and heavy ions, have attracted attention because the frequency and spectrum of the mutations induced by these particles are significantly different from those induced by electromagnetic radiation (Tanaka et al., 2010). For example, in carnation and chrysanthemum in which the mutations in color and shape of flowers were investigated after irradiation, carbon beam irradiation resulted in the higher mutation frequency and the wider mutation spectrum compared to gamma-irradiation (Okamura et al., 2003; Tanaka et al., 2010).

The type and energy of radiation determines the linear energy transfer (LET); that is, the amount of energy deposited to the encompassing material when an ionizing particle passes through a unit distance. Whole genome sequencing analyses of plant samples irradiated with gamma-rays, fast neutrons, and heavy ions, including carbon, argon, and iron ions, have been reported (Belfield et al., 2012; Hirano et al., 2015; Li et al., 2016, 2017; Shirasawa et al., 2016; Du et al., 2017; Kazama et al., 2017; Hase et al., 2018, 2020). The results from radiation with varying LETs showed that high-LET radiation (e.g., fast neutrons and heavy ion beams) induced more DNA structural variations (SVs; e.g., large deletions, inversions, translocations, and complex rearrangements) than low-LET radiation (e.g., gamma-rays), which induced mostly small-scale mutations, including single base substitutions (SBSs), and small insertions and deletions (InDels) (Jo and Kim, 2019). For example, argon ion beams (LET: 290 KeV μm^–1^) induced 4.4 times more SVs than carbon ion beams (LET: 30.0 KeV μm^–1^), but 2 times fewer small-scale mutations, including SBSs and small InDels, than carbon ion beams (Kazama et al., 2017). The type of DNA mutation is closely related to the impact of the mutation on gene function. Large SVs often result in deletion or truncation of genes, whereas SBSs and small InDels are usually associated with missense and frame-shift mutations, respectively. These relationships among LET, spectrum of DNA mutation, and impact on gene function are supported by the results of Kazama et al. (2008, 2011). They showed that ion beams with LET_*max*_ of 30.0 KeV μm^–1^ produced the highest phenotypic mutation rate among ion beams with various LETs, because radiation with this LET maximized null mutations of genes by inducing frequent SBSs and small InDels (Kazama et al., 2011). The unique relationship between LET radiation and types of DNA mutations can be explained by the mechanisms of DNA damage and repair. High-LET particles deposit energy to the target sample not only along the core track but also by projecting high-energy secondary electrons, forming a complex track structure (Ballarini et al., 2008). This track structure results in clustered DNA damages that include frequent DNA double-strand breaks (DSBs), which are difficult to repair. In contrast, low-LET radiation, which has multidirectional stochastic tracks scattered throughout the target, primarily damages DNA by producing super oxides that attack DNA and induce less frequent DSBs (Roots and Okada, 1975). Furthermore, Kazama et al. (2017) demonstrated that the primary type of non-homologous end joining (NHEJ) mechanism for DSB repair may differ according to the LET radiation dose rate.

Other factors also affect the frequency and/or spectrum of mutations. These include the irradiation dose, plant tissue type and condition, and irradiation period (Jo and Kim, 2019). Yamaguchi et al. (2009) showed that irradiation at the shoulder dose, from which the survival rate of plants from the irradiated seeds decreases rapidly according to increasing dose, maximized the number of phenotypic mutants in M_2_ generation per irradiated seeds. This relationship between irradiation dose and mutation frequency was applicable to gamma-rays and diverse ion beams. This finding is very useful for increasing the efficiency of radiation mutation breeding in which the appropriate irradiation dose is determined to increase mutation frequency and minimize other deleterious effects on survival and reproduction of plants. However, no whole-genome scale analysis that support this relationship between irradiation dose and mutation frequency have been reported so far.

Wilson (1946) was the first to propose the potential use of proton beams in cancer therapy. Since then, proton beams have received great attention because of their applicability to radiotherapy. Protons are low-LET (0.4–1.0 KeV μm^–1^ in 65–260 MeV proton beams used for therapy) until they are near the end of their path when they deposit most of their energy (Girdhani et al., 2013). These unique characteristics enabled targeted damaging of tumor tissues at specific depths. Besides the clinical applications, the biological effects of proton irradiation have been analyzed to predict the damage that may be caused by cosmic rays, which primarily consist of protons, on living organisms in space environment (Townsend, 2005). These studies focused on the potential toxicity of proton exposure to astronauts based on animal model experiments (Jäkel, 2008; Cengel et al., 2010). However, unlike for other ion beams, few studies have focused on the biological effects of proton beams in plants, although reports about the application of proton beam irradiation to plant mutagenesis are increasing (Kumar et al., 2018; Chauhan et al., 2019). Lee et al. (2015) showed that 45 MeV proton beams (1.461 KeV μm^–1^) induced many more DNA breaks than gamma-rays, and that 100 MeV proton beams (0.7306 KeV μm^–1^) caused more significant oxidative stress than gamma-rays in *Cymbidium*. The biological effects of proton beam irradiation on germination rate, shoot height, and plant weight have been investigated in soybean (Im et al., 2017). In addition, the frequency of small-scale mutations was estimated by genotype-by-sequencing in which short sequences that flanked the restriction sites of specific restriction enzymes were analyzed (Kim et al., 2018). However, comprehensive analysis of the frequency and spectrum of inheritable phenotypic mutations in M_2_ generation of plants and characterization of DNA mutations based on whole genome sequencing analysis have not been reported for proton beams in plants.

In this study, we characterized the mutagenic effect of proton beams by phenotypic and genomic investigations using the model plant Arabidopsis. Unique features of proton beam-induced mutations were detected by comparing them with the features of gamma-ray-induced mutations. The mutagenic effects on the phenotype and genome sequence also were compared between the two irradiations at different doses to obtain reasonable information for determining the optimal irradiation dose for mutation breeding. On the basis of the obtained results, we discuss the implications of proton beam-induced mutation on the plant DNA repair mechanism and the applicability of proton beam irradiation to plant mutagenesis.

## Materials and Methods

### Plant Materials and Irradiation Conditions

The seeds of *Arabidopsis thaliana* var. Landsberg *erecta* were provided by Kumho Life Science Laboratory (Kwangju Metropolitan City, Republic of Korea) and used as the plant material. Proton beams were irradiated using a proton linear accelerator (TR103) at the Korea Multi-purpose Accelerator Complex (KOMAC, Gyeongju, Korea), Korea Atomic Energy Research Institute. Dried Arabidopsis seeds were irradiated with the 100 MeV proton beam [LET = 0.7306 keV/μm] at 113.7, 190.3, 280.6, 393.4, 493.4, 574.0, 682.1, 786.9, 994.9, and 1,188.4 Gy. Gamma-ray irradiation was performed using a ^60^Co gamma-irradiator (150 TBq capacity; Atomic Energy of Canada Limited, Ottawa, Canada) at the Advanced Radiation Technology Institute (ARTI, Jeongeup, Korea), Korea Atomic Energy Research Institute. Dried Arabidopsis seeds were irradiated with gamma-rays [LET = 0.2 keV/μm] at 200, 400, 600, 900, 1200, and 1500 Gy.

### Analysis of the Effect of Irradiation on Survival Rates (M_1_ Generation) and Phenotypes (M_2_ Generation)

The irradiated Arabidopsis seeds were sown in soil and cultivated at 22°C under a 16-h light/8-h dark photoperiod. Survival rate was determined 4 weeks after seeding using four replications with 50 seeds. The LD_50_ dose (50% of the plants die) and the shoulder dose (*Dq*) were obtained from the survival rate–irradiation dose curve. To calculate the shoulder dose, we used a single-hit multitarget equation (Hase et al., 2012) as follows.


$$S\,u\,r\,v\,i\,v\,a\,l\,r\,a\,t\,e=1-{\left(1-e^{-D/D_{0}}\right)}^{m}$$

where *D* is the dose, *D*_0_ is the dose that results in 37% survival rate, and *m* is the extrapolating number, which was calculated based on the least-squares method. The shoulder dose was calculated according to Hase et al. (2020) as follows.


$$D_{q}=D_{0}\times I\,n\,m$$

To investigate the phenotypic mutations, the seeds of 362–490 M_1_ lines were obtained from each proton beam irradiation at 493.4, 682.1, and 994.9 Gy, and gamma-irradiation at 600, 900, and 1,200 Gy. One M_2_ individual from each M_1_ line was grown on plates (100 × 30 mm) with 70 ml of 0.5 × MS medium (2% sucrose, 0.6% plant agar, 0.05% MES, pH 5.7). In each plate, seven M_2_ individuals from different M_1_ lines were grown together. Three weeks after seeding, mutants were identified by screening for color, shape, and developmental characteristics of the leaves.

### Whole Genome Sequencing and Analysis of DNA Mutations

Six individuals were randomly selected from the M_2_ lines obtained from the proton beam irradiations at 493.4, 682.1, and 994.9 Gy, and the gamma-ray irradiation at 900 Gy. DNA was extracted from the rosette leaves of each individual plant using a cetyltrimethylammonium bromide (CTAB)-based method (Cota-Sánchez et al., 2006). DNA sequencing to obtain paired-end reads (100 bp or 150 bp) was performed for the 24 selected M_2_ plants and two wild-type individuals using the Illumina HiSeq 2000 or HiSeq X Ten (Illumina, Inc., San Diego, CA, United States) platform that was commercially available at Theragen Etex (Seongnam-si, Republic of Korea). For sequence pre-processing, PCR duplicate reads were removed from the raw sequences and quality trimming was performed to obtain sequences that contained at least 25 high-quality bases (phred score ≥ 20) using the SolexaQA (v.1.13) package (Cox et al., 2010). The cleaned reads were mapped to the L*er* assembly as the reference genome (GenBank accession number: GCA_001651475) using the BWA (0.6.1-r104) program (Li and Durbin, 2009) to generate BAM format files. SBSs and small InDels (<100 bp) between the reference genome and the mapped sequences from each sample were called from the BAM format files using SAMtools (0.1.16) (Li et al., 2009), an in-house script (Kim et al., 2014), and Pindel (0.2.5) (Ye et al., 2009). For SAMtools, the minimum mapping qualities for SBSs and gaps were set as 30 and 15, respectively, and the read depth range was set as 10–505. The SBSs and InDels from each sample were integrated to generate an SBS and InDel matrix to detect variations between samples. If the proportion of mutant reads was ≥80% in a mutation site, the mutation was defined as homozygous; if the proportion was ≥25 and <80%, the mutation was defined as heterozygous. To detect SVs, duplicate sequences were removed from the BAM format files using the Picard MarkDuplicates program^1^. Then, BreakDancer (version 1.4.5) (Chen et al., 2009) was used to detect SVs. For BreakDancer, the default settings were used, except the minimum number of read pairs required to establish a connection was set as 3. The SVs from each sample were integrated to generate an SV matrix to detect variations between samples. The SBSs, small InDels, and SVs that were common between the wild-type individuals but were polymorphic between wild-type individuals and a single M_2_ line were selected and used for the analysis. Mutations that were commonly detected in two or more M_2_ lines were excluded.

### Validation of SVs and Reconstruction of SV Formation Processes

The SVs detected using BreakDancer were visualized and explored using the Integrative Genome Viewer (IGV; Robinson et al., 2011). To validate the SVs, we designed primers from the flanking sequences of each SV junction and performed PCRs to determine whether the amplicons expected from each SV model were generated. The sequence of each amplicon was obtained by Sanger sequencing. BLAST was used to align the amplicon sequences from the wild type and M_2_ lines that contained the SVs to determine the SV junction position and reconstruct the SV formation processes.

## Results

### Effect of Proton Beam and Gamma-ray Irradiation on Survival Rate of Arabidopsis

**FIGURE 1:**
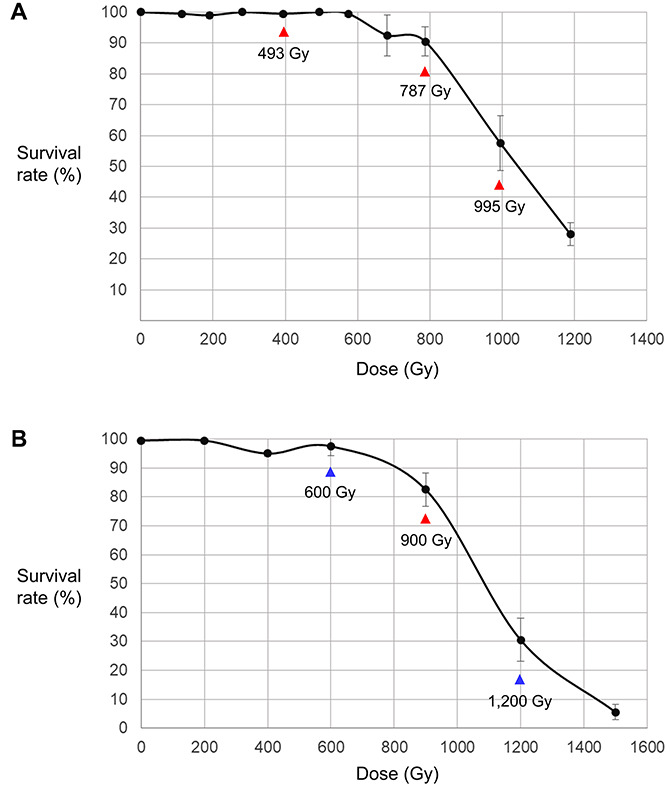
Relationship between irradiation dose and survival rate of M_1_ individuals. **(A)** Dose–survival rate relationship in proton beam-irradiated individuals. **(B)** Dose–survival rate relationship in gamma-ray-irradiated individuals. The samples irradiated at the doses indicated by red arrows were used for both the phenotypic analysis and whole genome sequencing. The samples irradiated at the doses indicated by blue arrows were used only for the phenotypic analysis.

The survival rates of Arabidopsis were investigated 4 weeks after sowing dry seeds irradiated with 100 MeV proton beams (LET = 0.7306 keV/μm) or gamma-rays (LET = 0.2 keV/μm) at different irradiation doses (Figure 1). In both analyses, high survival rates were maintained at low doses without significant change, but they decreased rapidly at doses higher than the dose that was defined as the shoulder dose (*Dq*) in previous studies (Yamaguchi et al., 2009; Hase et al., 2018). The *Dq* calculated following Hase et al. (2012) (see section “Materials and Methods”) was 13% lower for proton beam irradiation (754 Gy) than it was for gamma-ray irradiation (860 Gy), which indicates that the negative effect of proton beams on plant survival was slightly higher than that of gamma-rays. The LD_50_ was also slightly lower for the proton beam irradiation (1,051 Gy) than it was for the gamma-ray irradiation (1,086 Gy). Among the irradiation doses used in this study, we selected the doses that were closest to LD_50_, *Dq*, and two-thirds of *Dq*; namely, 995, 787, and 493 Gy for proton beams and 1,200, 900, and 600 Gy for gamma-rays, respectively. The M_2_ lines developed from the irradiations at these doses were used for the subsequent mutation analysis.

### Frequency and Spectrum of Phenotypic Variations in M_2_ Mutation Populations

**FIGURE 2:**
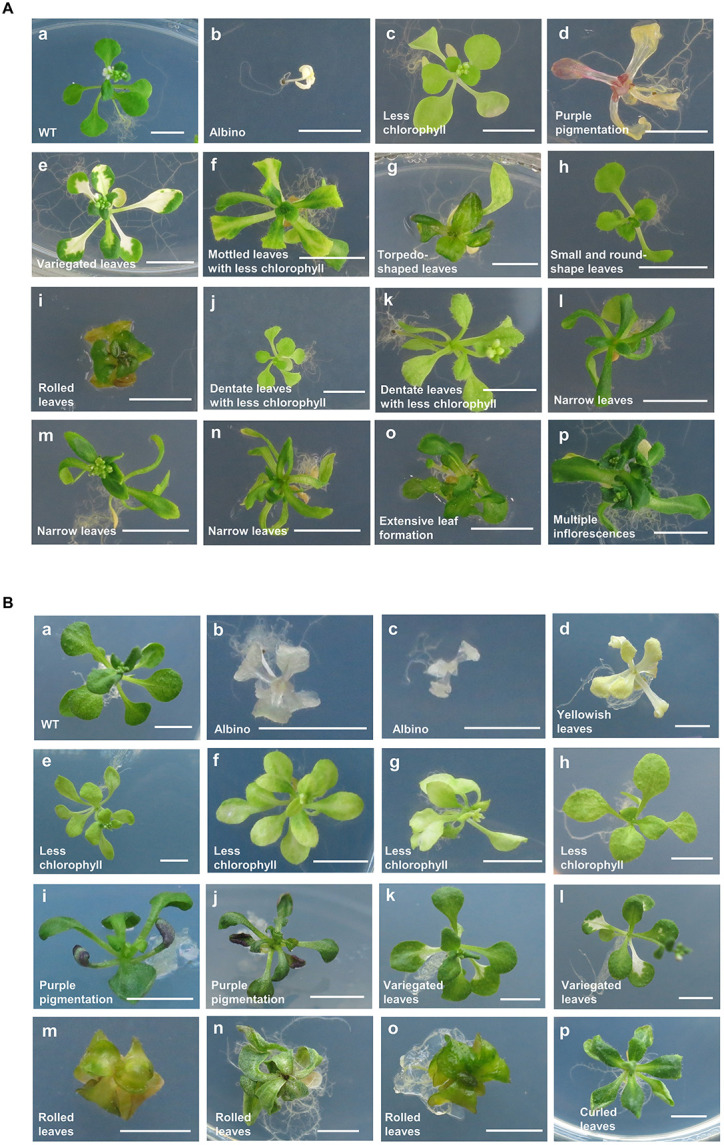
Representative mutant phenotypes detected in M_2_ populations. Mutant phenotypes were classified in M_2_ populations derived from proton beam **(A)** and gamma-ray **(B)** irradiation. Images were taken on plates 22 days after sowing. Scale bars, 0.5 cm.

**FIGURE 3:**
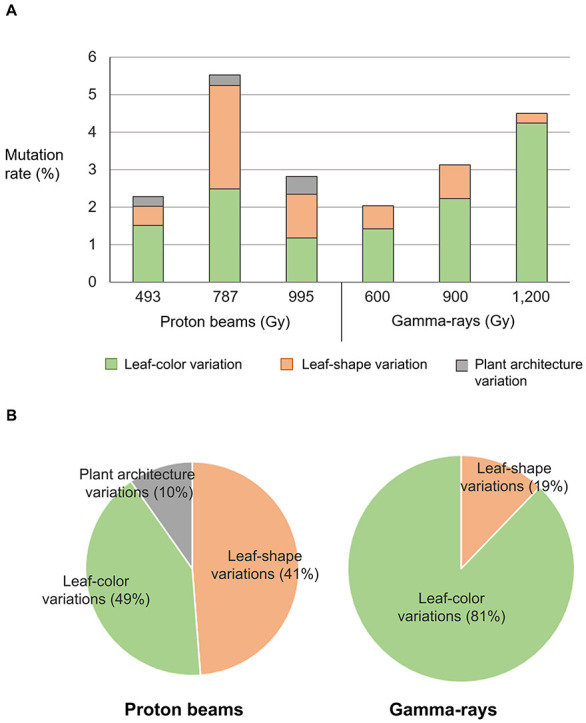
Classification of mutant phenotypes detected in M_2_ lines derived from proton beam and gamma-ray irradiation. **(A)** Mutation frequency in M_2_ lines classified by types of mutant phenotypes. **(B)** Ratio between mutation frequencies of M_2_ lines classified by types of mutant phenotypes.

Phenotypes were investigated visually 3 weeks after seeding in the M_2_ mutant populations developed by proton beam (493, 787, and 995 Gy) or gamma-ray (600, 900, and 1,200 Gy) irradiation. The detected mutant phenotypes were categorized as changes in leaf color, leaf shape, and plant architecture (Table 1 and Figure 2). The highest mutation rate obtained among the proton beam populations (5.52%) was slightly higher than that obtained among the gamma-ray populations (4.50%) (Table 1 and Figure 3A). The dose (787 Gy) near the shoulder dose (*Dq*) for proton beam irradiation produced the highest mutation frequency, whereas the highest dose (1,200 Gy) near the LD_50_ for gamma-ray irradiation produced the highest mutation frequency. Compared to the highest mutation frequencies obtained in each radiation source, the mutation frequencies for the lowest doses that corresponded to two-thirds of *Dq* were more than two times lower for proton beam irradiation (2.28% in 494 Gy-irradiated population) and gamma-ray irradiation (2.04% in 600 Gy-irradiated population). The proton beams and gamma-rays also produced a different mutation spectrum. Most of the mutations (81%) found in the gamma-ray populations resulted in leaf color variations, such as albino, light green, purple, yellowish, variegated, and mottled leaves (Table 1 and Figures 2B, 3B). Proton beams produced broader and a more evenly distributed mutation spectrum than gamma-rays (Table 1 and Figures 2A, 3B). Several mutant characteristics of leaf shape (narrow, torpedo-shaped, and curled leaves) and plant architecture (extensive vegetative growth and formation of multiple main inflorescences) were detected only in the proton beam-irradiated populations. In addition, the frequencies of leaf-shape (41%) and leaf-color (49%) mutations induced by proton beam irradiation were similar between these two phenotypic categories.

**TABLE 1:** Mutant phenotypes in M_2_ populations of Arabidopsis irradiated with proton beams or gamma-rays.

**Classification of mutants**	**Characteristics**	**Non-irradiated**	**Proton beam-irradiated (Gy)**			**Gamma-irradiated (Gy)**				
			**494**	**787**	**995**	**Total**	**600**	**900**	**1,200**	**Total**
Leaf color mutants	Albino		2	4	1	7	1	2	4	7
	Low chlorophyll		3	2	2	7	5	4	9	18
	Purple pigmentation			1	2	3		3	3	6
	Yellowish					0	1			1
	Variegated		1	1		2		1	1	2
	Mottled			1		1				
	Total	0	6	9	5	20	7	10	17	34
Leaf shape mutants	Narrow		1	1		2				0
	Round			1		1		2		2
	Torpedo-shaped		1			1				0
	Curled				1	1				0
	Rolled			5	1	6		2	1	3
	Dentate			3	2	5	2			2
	Larger				1	1	1			1
	Total	0	2	10	5	17	3	4	1	8
Other mutants	Extensive leaf formation		1		2	3				0
	Multiple main inflorescences			1		1				0
	Total	0	1	1	2	4	0	0	0	0
	Mutation rate (%)	0	9/395 (2.28)	20/362 (5.52)	12/426 (2.82)	41/1183 (3.47)	10/490 (2.04)	14/447 (3.13)	18/400 (4.50)	42/1337 (3.14)

### Mutation Frequency Detected by Whole Genome Sequencing

**FIGURE 4:**
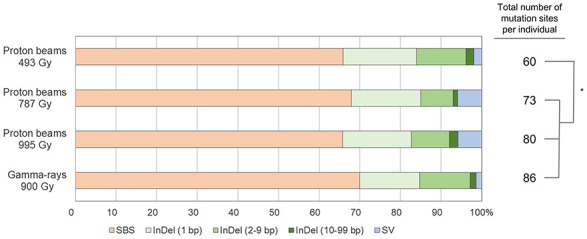
Relative frequency and spectrum of DNA mutations induced by proton beam and gamma-ray irradiation. The asterisk indicates significant differences between groups (*p* < 0.05 in student’s *t*-test).

Whole genome sequencing was performed for six individual M_2_ plants derived from irradiations using 100 MeV proton beams at 493, 787, and 995 Gy, and gamma-rays at 900 Gy, respectively (hereafter, each M_2_ plant group is referred to as “P493,” “P787,” “P995,” and “G900,” respectively). The generated sequencing reads were mapped on the 97.8% DNA region (on average) of the reference genome. The sequencing depth of the mapped reads was 46.6× on average (Supplementary Table 1). DNA mutations detected by comparative analysis between non-irradiated plants (two individuals) and each irradiated individual were classified into three main groups, SBSs, small InDels (<100 bp), and SVs, including large deletions (≥100 bp), duplications, inversions, and translocations. The average numbers of total mutated sites were 60, 73, 80, and 86 in P493, P787, P995, and G900, respectively (Figure 4). The numbers were significantly higher (*p* < 0.05) in P787, P995, and G900 than in P493, but no significant difference was detected among the numbers in P787, P995, and G900. This result indicates that the mutation frequency was not greatly changed when the proton beam irradiation dose was increased from *Dq* to LD_50_ or when the radiation source was changed from proton beams to gamma-rays, whereas lowering the proton beam irradiation dose below *Dq* more affected mutation frequency. In each plant group, the proportion of mutation sites with SBSs was commonly highest (65.8–70.0%) among the three types of mutation sites. The total number of DNA regions with rejoined junctions of SVs was 26 and 28 in the six individuals of P787 and P995, respectively, but only 7 in the six individuals of both P494 and G900 (Figure 4). Therefore, the proton beam irradiation induced more SVs than the gamma-ray irradiation at doses that resulted in similar survival rates, and changing from the lower irradiation dose to *Dq* increased the number of SVs produced by proton beam irradiation.

### Characteristics of SBSs and Small InDels

**FIGURE 5:**
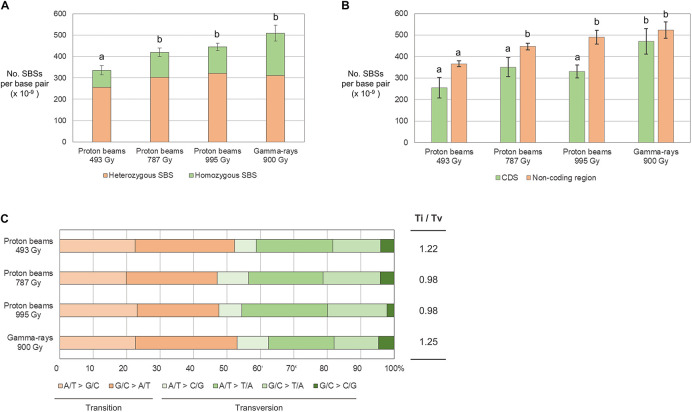
Frequency and types of single base substitutions (SBSs) in M_2_ lines derived from proton beam and gamma-ray irradiation. **(A)** Frequency of SBSs classified according to zygosity. **(B)** Frequency of SBSs classified according to their location on protein coding (CDS) and non-coding DNA regions. **(C)** Proportions of SBSs classified according to the types of changed nucleotides. Different letters above the bars indicate statistical differences determined by one-way ANOVA followed by Duncan’s multiple range test for classification criteria among the irradiation groups. Ti/Tv is the ratio between frequency of transitions and transversions.

The frequency of SBSs in each group was between 3.35 × 10^–11^/bp and 5.09 × 10^–11^/bp. The frequency of SBSs in P494 was significantly lower than those in the other three groups, and no significant difference was detected among those in the three groups (Figure 5A). The frequency of homozygous SBSs was highest in G900, followed by P995, P787, and P494 (Figure 5A). The heterozygous-to-homozygous ratio for SBSs ranged from 0.31 in P494 to 0.64 in G900. However, variations in this ratio among individuals was quite high, and therefore no significant difference (*p* < 0.05) from the ratio theoretically expected based on Mendelian segregation (0.5) was detected in any of the groups. The frequency of SBSs in the protein coding regions was lower than that in the non-coding regions in P995, whereas significant difference was not detected between the frequency of SBSs in the coding and non-coding regions in P493, P787, and G900 (Figure 5B). The transition-to-transversion ratio was relatively lower in the proton beam-irradiated groups (0.98–1.22) than it was the gamma-ray-irradiated group (1.25) (Figure 5C).

**FIGURE 6:**
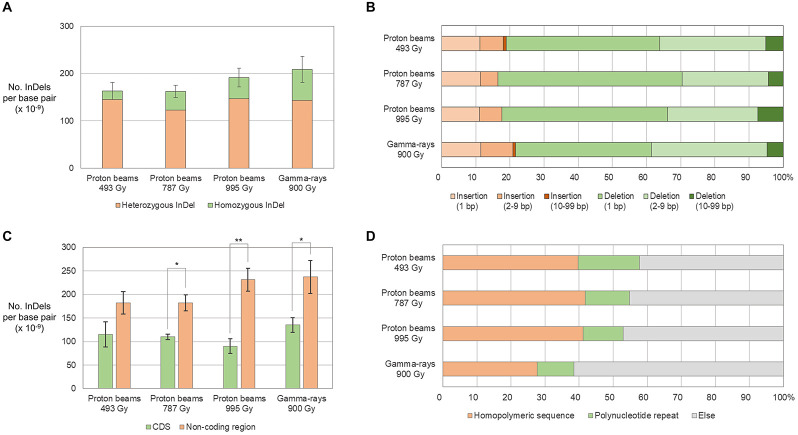
Frequency and types of small InDels (<100 bp) in M_2_ lines derived from proton beam and gamma-ray irradiation. **(A)** Frequency of small InDels classified according to zygosity. **(B)** Proportions of small InDels classified according to their length. **(C)** Frequency of small InDels classified according to their location on protein coding (CDS) and non-coding DNA regions. **(D)** Proportions of small InDels classified according to the surrounding repeat sequences. Asterisks indicate significance of differences between groups (*p < 0.05, **p < 0.05 in student’s *t*-test). Ti/Tv is the ratio between frequency of transitions and transversions.

The frequency of small InDels (<100 bp) was between 1.62 × 10^–11^/bp (P787) and 2.08 × 10^–11^/bp (G900) and was not significantly different between plant groups (Figure 6A). However, the frequency of homozygous InDels was significantly higher in G900 than it was in P493 because the homozygous-to-heterozygous ratio for InDels was higher in G900 (0.45) than it was in P493 (0.13) (Figure 6A). Deletions were from 3.63 (G900) to 5.04 (P787) times more frequent than insertions (Figure 6B). By length, 1-bp deletions accounted for the largest proportion of InDels (from 39.9% in G900 to 53.9% in P787) in all the plant groups, followed by 2–9-bp deletions (between 25.2% in P787 to 33.8% in G900) (Figure 6B). Small InDels were more frequently detected in non-coding regions than in protein coding regions in P493, P995, and G900 (Figure 6C). Previous research showed that homopolymeric sequences or polynucleotide repeats were often found at the junction of small deletions (Belfield et al., 2012; Du et al., 2017; Hase et al., 2018). This is consistent with our finding that homopolymeric sequences and polynucleotide repeats were found at 27.7–41.7% and 10.8–18.1% of the junctions, respectively (Figure 6D).

### Characteristics of SVs

**FIGURE 7:**
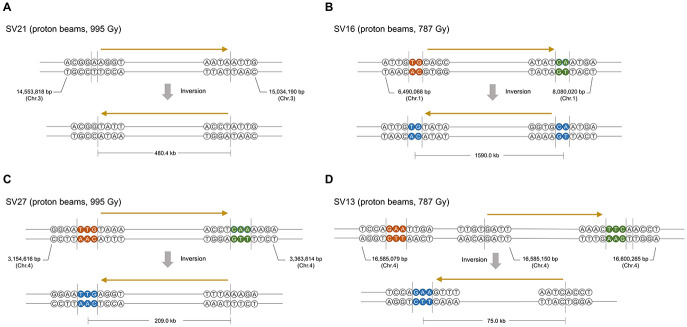
Representative typesFigure of inversions induced by proton beam irradiation. **(A)** Inversion without microhomologous sequences and deletion on rearrangement junctions. **(B)** Inversion with microhomologous sequences and without deletion. **(C)** Inversion with microhomologous sequences and deletion of a few nucleotides near or on microhomologous sequences. **(D)** Inversion with microhomologous sequences and deletion of 10 s to 100 nucleotides on the adjacent region of rearrangement junctions. Brown and green circles indicate the microhomologous sequences on each rearrangement site in the original molecule. Blue circles in the inversion products indicate nucleotides that may have originated from any microhomologous sequences in the original molecule. The ranges of DNA regions in which DNA breaks or end-joining could occur are indicated by vertical lines. The relative direction of the DNA region is indicated by arrows above the DNA sequences.

We predicted SVs by *in silico* analysis of the short read sequences mapped on the reference genome (see section “Materials and Methods”) and validated them by PCR amplification followed by Sanger sequencing of the amplicons. Information about types, zygosity, and position in the genome was obtained in 39 rearrangement processes that were expected from the identified SVs (Table 2, Supplementary Table 3, and Supplementary Figures 1, **2**). Only four and five rearrangement processes were expected in G900 and P494, respectively, whereas 15 were expected in both P787 and P995 (Table 2). Unlike heavy ion beams that easily generate complex DNA rearrangement by inducing several rearrangement events simultaneously in a short DNA region (Kazama et al., 2017), the proton beams and gamma-rays mostly induced rearrangement process that were expected to involve a single DNA rearrangement event such as deletion, inversion, translocation, and duplication. Only two SV events, SV18 and SV22 (Supplementary Table 3 and Supplementary Figure 2), involved multiple rearrangement events, which were two inversions. The 10 and 31 rearrangement events were homozygous and heterozygous, respectively. The size of the deleted or inverted DNA fragment was relatively small (<20 kb) in most of homozygous events, whereas it often exceeded 100 kb in heterozygous events (Supplementary Table 3). Inversions accounted for the largest portion (23 of 41) among the different types of rearrangement events, followed by large deletions (12 of 41) (Table 2, Supplementary Table 3). Most inversions (20 of 23) contained at least a rearrangement junction, which was expected to be formed by joining of the end sequences with microhomology between them (2–23 bp in size) (Table 3, Figure 7, and Supplementary Table 3 and Supplementary Figure 2). Although the overlapped sequences were maintained after rearrangements in three of the inversions, they were partially deleted in 17 other inversions (Figure 7C, Supplementary Table 4). In addition to deletion on the overlapped sequences, 11 inversions were associated with deletion (1–61 bp in length) on the adjacent DNA regions of the overlapped sequences (Figure 7D, Supplementary Table 4).

**TABLE 2:** Structural variations in M_2_ mutants of Arabidopsis irradiated with proton beams or gamma-rays.

**Sample ID**	**Type**	**Location of DNA breaks**		**Size of inversed or deleted or duplicated fragment (kb)**	**Zygosity of structural variations (SVs)**	**Number of truncated genes**	**Number of deleted genes**
		**Break point 1**	**Break point 2**				
		**Chromosome number (location; kb)**	**Chromosome number (location; kb)**				
P493-1^*z*^	Inversion	1 (6150.1)	1 (7404.1)	1254.0	Heterozygous	2	0
P493-1	Deletion	3 (6848.9)	3 (6868.1)	19.2	Homozygous	0	4
P493-4	Deletion	3 (2675.7)	3 (2742.3)	66.6	Heterozygous	2	20
P493-5	Inversion	5 (24769.6)	5 (25113.1)	343.6	Heterozygous	2	0
P493-6	Deletion	3 (16383.6)	3 (16559.3)	176.7	Heterozygous	0	47
P787-1	Deletion	5 (22897.2)	5 (22909.6)	12.4	Heterozygous	2	4
P787-2	Inversion	4 (8941.6)	4 (9211.0)	269.2	Heterozygous	0	0
P787-2	Deletion	5 (19413.2)	5 (19416.9)	3.7	Homozygous	1	1
P787-3	Inversion	1 (28507.0)	1 (29353.5)	846.4	Heterozygous	1	0
P787-3	Inversion	2 (12656.5)	2 (13216.5)	560.0	Heterozygous	1	0
P787-3	Deletion	2 (13166.0)	2 (13635.5)	469.5	Heterozygous	1	127
P787-3	Interchromosomal translocation	2 (2385.0)	5 (5354.5)	874.7	Heterozygous	0	0
			5 (6229.2)^y^		Heterozygous	0	0
P787-3	Inversion	5 (16585.1)	5 (16660.3)	75.1	Heterozygous	1	0
P787-4	Deletion	2 (3072.1)	2 (3072.9)	0.8	Heterozygous	1	0
P787-5	Inversion	5 (24670.0)	5 (25022.4)	352.4	Heterozygous	1	0
P787-6	Inversion	1 (6490.1)	1 (8080.0)	1589.9	Heterozygous	1	0
P787-6	Inversion	1 (13595.3)	1 (13793.2)	197.9	Heterozygous	0	0
P787-6	Inversion	4 (7403.4)	4 (11612.1)	4208.7	Heterozygous	1	0
		4 (11612.1)^x^	4 (11612.9)	0.7	Heterozygous		
P787-6	Deletion	5 (12250.2)	5 (12250.7)	0.5	Heterozygous	0	0
P787-6	Interchromosomal translocation	1 (24564.6)	4 (13145.3)		Heterozygous	2	0
P995-1	Inversion	3 (14553.8)	3 (15034.2)	480.4	Homozygous	1	0
P995-1	Inversion	4 (9114.6)	4 (9436.6)	322.0	Heterozygous	1	0
		4 (9114.6)^x^	4 (9718.0)	603.4	Heterozygous	1	80
P995-1	Interchromosomal translocation	4 (2320.8)	5 (21780.2)		Heterozygous	0	0
P995-2	Deletion	2 (6777.8)	2 (6799.4)	21.6	Heterozygous	0	2
P995-4	Inversion	2 (8396.2)	2 (9163.3)	767.1	Heterozygous	1	0
P995-4	Inversion	3 (13989.4)	3 (18192.8)	4203.4	Homozygous	0	0
P995-4	Inversion	4 (3154.6)	4 (3363.6)	209.0	Heterozygous	0	0
P995-4	Inversion	4 (14934.7)	4 (15407.4)	472.7	Heterozygous	1	0
P995-4	Inversion	5 (10530.7)	5 (10863.8)	333.1	Heterozygous	0	0
P995-4	Inversion	5 (20353.5)	5 (20843.1)	489.6	Homozygous	1	0
P995-4	Deletion	5 (20983.7)	5 (20986.5)	2.7	Homozygous	0	0
P995-4	Deletion	5 (22528.3)	5 (22549.4)	21.2	Homozygous	1	4
P995-4	Interchromosomal translocation	5 (13499.0)	scaffold15_ Contig624 (84.6)		Heterozygous	1	0
P995-5	Inversion	1 (320.3)	1 (723.9)	403.6	Heterozygous	2	0
P995-6	Interchromosomal translocation	1 (2866.4)	5 (4681.7)	3.6	Heterozygous	0	0
G900-1	Inversion	1 (11198.8)	1 (11203.4)	5.6	Homozygous	2	0
G900-1	Interchromosomal translocation	3 (1725.5)	4 (8909.7)		Homozygous	1	0
G900-2	Inversion	3 (2352.9)	3 (2353.6)	0.7	Homozygous	0	0
G900-5	Deletion	2 (14586.0)	2 (14599.6)	13.6	Heterozygous	1	1

*^*Z*^ In sample IDs, “P” and “G” indicate proton beam and gamma-ray irradiation, respectively. The numbers indicate the irradiation dose (Gy) and sample number. Two inversions detected in a rearrangement process were represented together.*

*^*y*^ Because interchromosomal duplications were expected in this case, three DNA break points, two for the duplicated fragment and one for the inserted site, were predicted (see Supplementary Figure 1).*

**TABLE 3:** Classification of irradiation-induced inversions according to the characteristics of sequences near the junctions of inverted DNA regions.

**Radiation Source (dose; Gy)**	**Inversion with overlapped sequence**	**Deletion on overlapped sequence**	**Insertion on overlapped sequence**	**Deletion at adjacent area of overlapped sequence**	**Insertion at adjacent area of overlapped sequence**	**Total number of inversions**
Proton beams (493 Gy)	2 (100 %)	2 (100%)	0	0	0	2
Proton beams (787 Gy)	8 (89%)	6 (67%)	1 (11%)	2 (22%)	1 (11%)	9
Proton beams (995 Gy)	8 (80%)	7 (70%)	0	5 (50%)	0	10
Gamma-rays (900 Gy)	2 (100%)	2 (100%)	0	1 (50%)	0	2
						

### Effect of DNA Mutations on Gene Functions

Mutations in the protein coding sequences were analyzed to predict the impact of DNA mutations on gene function. The total number of genes for which the sequences or lengths of the encoding proteins were changed by DNA mutations was highly variable among individuals because heterozygous large deletions in several individuals resulted in the loss of many genes at once. This hampered statistical analysis to determine significant differences between groups. Because most of the deleted fragments in the homozygous SVs were relatively short and contained fewer genes, variation in the number of genes affected by homozygous mutations among individuals was relatively small. The average number of genes affected by homozygous mutations was highest in G900 (5.33 genes) and lowest in P494 (3.33 genes) (Table 4). When genes affected by SVs were excluded, the average number of genes affected by homozygous or heterozygous mutations was higher in G900 (14.5 genes) and P787 (12.7 genes) compared with P494 (9.3 genes) (Table 4), and the average number of genes with homozygous mutations ranged from 2.7 (P493) to 4.2 (P787) and 4.8 (G900). Our results indicated that proton beam or gamma-ray irradiation at *Dq* induced mutations in a similar number of genes, if SVs were excluded. The proportion of genes affected by high impact mutations was 31.6% in P787 and 27.6% in G900, and 36.0% in P787 and 20.7% in G900 for only homozygous mutations, when genes affected by SVs were excluded from the analysis (Table 4). When the effect of SVs was included, the average number of genes with high impact mutations was much higher in P787 (28.2 genes) than it was in G900 (4.8 genes), but most of these genes carried heterozygous mutations and the variation among individuals was very high.

**TABLE 4:** Number of genes affected by DNA mutations induced by proton beams and gamma-rays.

**Significance of impact**	**Mutation type**	**Proton beams (493 Gy)**	**Proton beams (787 Gy)**	**Proton beams (995 Gy)**	**Gamma-rays (900 Gy)**
Moderate impact mutation	Missense	5.33 ± 0.81^*z*^ (2.17 ± 0.60)^*y*^	8.33 ± 0.84 (2.5 ± 0.66)	7.33 ± 1.12 (2.17 ± 0.15)	9.33 ± 1.43 (3.83 ± 0.98)
	In-frame deletion	1.00 ± 0.24 (0.17 ± 0.15)	0.33 ± 0.19 (0.17 ± 0.15)	0.67 ± 0.3 (0)	0.83 ± 0.28 (0)
	In-frame insertion	0	0	0	0.33 ± 0.19 (0)
	Total of moderate impact mutations	6.33 ± 0.81 (2.34 ± 0.51)	8.66 ± 0.9 (2.67 ± 0.65)	8 ± 1.13 (2.17 ± 0.15)	10.49 ± 1.45 (3.83 ± 0.98)
High impact mutation	Start codon loss	0	0.33 ± 0.19 (0)	0	0.17 ± 0.15 (0.17 ± 0.15)
	Premature stop codon generation	0.17 ± 0.15 (0)	0.33 ± 0.30 (0)	0.33 ± 0.19 (0.33 ± 0.19)	0.50 ± 0.20 (0)
	Frame shift	2.83 ± 0.83 (0.33 ± 0.19)	3.33 ± 0.30 (1.50 ± 0.31)	2.33 ± 0.56 (0.5 ± 0.31)	3.33 ± 0.45 (0.83 ± 0.44)
	Truncation of gene	1.00 ± 0.41 (0)	2.17 ± 0.55 (0.17 ± 0.15)	1.50 ± 0.74 (0.50 ± 0.31)	0.67 ± 0.45 (0.50 ± 0.46)
	Deletion of complete gene	11.83 ± 7.04 (0.67 ± 0.61)	22.00 ± 19.18 (0.17 ± 0.15)	1.00 ± 0.62 (0.67 ± 0.61)	0.17 ± 0.15 (0)
	Total high impact mutations	15.83 ± 7.27 (1.00 ± 0.58)	28.16 ± 20.07 (1.84 ± 0.5)	5.17 ± 1.36 (2.00 ± 0.82)	4.84 ± 0.68 (1.50 ± 0.61)
	Total high impact mutations excluding mutations induced by structural variations	3.00 ± 0.85 (0.33 ± 0.19)	4.00 ± 0.62 (1.50 ± 0.31)	2.67 ± 0.56 (0.83 ± 0.44)	4.00 ± 0.33 (1.00 ± 0.41)
Silent mutation		3.17 ± 0.89 (0.83 ± 0.15)	2.67 ± 0.69 (0.83 ± 0.28)	3.67 ± 0.51 (0.83 ± 0.28)	4.50 ± 0.91 (1.67 ± 0.45)
Total number of mutated genes(excluding silent mutations)		22.17 ± 7.71 (3.33 ± 0.90)	36.83 ± 20.15 (4.50 ± 0.70)	13.17 ± 1.88 (4.17 ± 0.76)	15.33 ± 1.15 (5.33 ± 1.24)
Total number of mutated genes(excluding silent mutations and mutations induced by structural variations)		9.33 ± 1.56 (2.67 ± 0.38)	12.67 ± 0.96 (4.17 ± 0.72)	10.67 ± 1.39 (3.00 ± 0.41)	14.50 ± 1.45 (4.83 ± 1.28)

*^*z*^ Average number of affected genes carrying homozygous or heterozygous mutations ± standard error.*

*^*y*^ Value in parenthesis is the average number of affected genes carrying homozygous mutations ± standard error.*

## Discussion

### Unique Mutagenic Effect of Proton Beams

As a major component of cosmic-rays, protons may have affected the evolution of plants on Earth and could influence the growth and inheritance of plants that will be cultivated in extraterrestrial space environments in the coming space era (Girdhani et al., 2013). Protons can also be used as a novel mutagen in plant mutation breeding as other radio-active particles such as heavy ion beams and fast neutrons, which have been widely applied in breeding after characterization at the molecular level (Kim et al., 2018). We performed a comprehensive study using both phenotype and whole genome sequence analyses to characterize the mutagenic effects of proton beams in plants.

The phenotype analysis showed that proton beam irradiation resulted in a broader range of mutation spectrum than gamma-ray irradiation when the irradiation dose was *Dq* or LD_50_, although the mutation frequency was not clearly different between the two irradiation sources. The reason for the broader mutation spectrum is not clear, but the higher proportion of genes carrying high impact mutations and the higher number of SVs produced in the proton beam irradiation may be causal factors. The high impact mutations, such as complete loss or truncation of genes and generation of premature stop codons, are likely to induce loss-of-function of genes that may result in visually detectable distinctive phenotypic changes. In addition, SVs, such as large deletions or inversions, not only directly affect the copy number or structure of genes but also may change the genomic landscape that is related to the transcription pattern of genes in specific genomic areas (Fransz et al., 2016). Alonge et al. (2020) showed that natural SVs led to changes in important traits in tomato (fruit size, flavor, production) by modifying gene expression levels and dosage. In peach, Guan et al. (2021) reported that a 1.67 Mb inversion lead to upregulation of *PpOFP2* gene located nearby an inversion junction, and resulted in a change of fruit shape. The impact of SVs on gene expression has been studied more deeply in humans. Alterations in the genomic location of genes by inversions or translocations were suggested to cause position effects by changing the epigenetic states of DNA regions near break points (Genesio et al., 2011; Finelli et al., 2012). In such cases, the combinational effects of the multiple genes that were affected at the transcriptional levels may lead to phenotypic changes that cannot be induced by a single gene mutation. Carbon ion beam irradiation, which can also induce frequent SVs, resulted in a broader spectrum of mutant phenotypes in chrysanthemum (Tanaka et al., 2010) and carnation (Okamura et al., 2003) than that obtained with gamma-ray irradiation.

**FIGURE 8:**
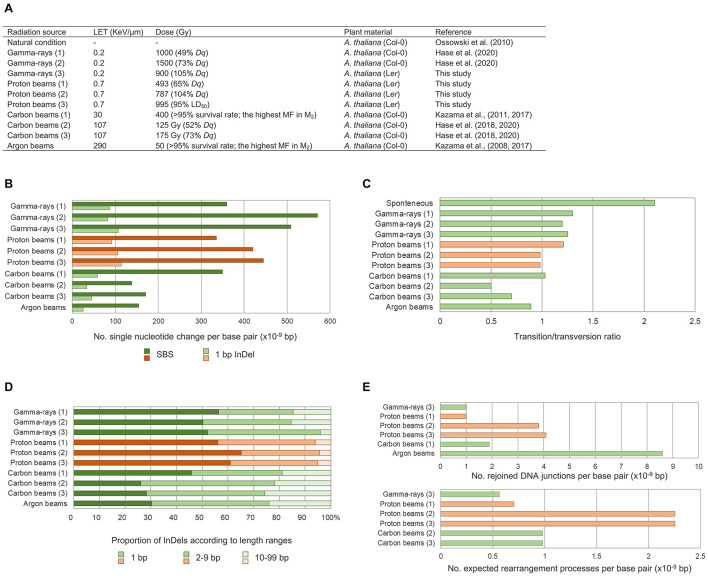
Comparison of mutagenic effects according to radiation sources and irradiation doses. **(A)** Irradiation conditions and samples in this study and in previous studies that used Arabidopsis M_2_ lines. **(B)** Frequency of single nucleotide variations including single base substitutions (SBSs) and 1-bp deletions. **(C)** Transition-to-transversion ratios. **(D)** Proportions of small InDels classified according to their sizes. **(E)** Frequency of structural variations (SVs). The number of rejoined DNA junctions found in this study were compared with the number found by Kazama et al. (2017), and the number of expected rearrangement events found in this study were compared with the number found by Hase et al. (2018, 2020) so that the data could be used as they were represented in each of the previous studies. Red and green bars represent plant groups derived from proton beam irradiation and other types of irradiations, respectively.

The comparative analysis between the whole genome sequences of Arabidopsis M_2_ lines derived by proton beam and gamma-ray irradiation in this study and by heavy ion beam irradiation in previous studies (Kazama et al., 2017; Hase et al., 2018, 2020) enabled characterization of the mutagenic effects according to the LET or types of radiation (Figure 8). Relatively more frequent single nucleotide mutations (SBS and 1-bp InDels) were detected with low-LET radiation (gamma-rays and proton beams) than with high-LET radiation (carbon and argon beams). In addition, the transition-to-transversion ratio and proportion of InDels < 10 bp among the small InDels (<100 bp) were generally higher with the low-LET radiation. Unlike these patterns, the number of SVs was expected to be affected not only by LET but also by the type of radiation. Proton beam irradiation induced notably more frequent SVs than those produced by gamma-ray irradiation when both irradiations were performed at irradiation doses (*Dq*) that caused similar impairment in plant survival. The frequency of SVs induced by proton beam irradiation at *Dq* was higher than that produced by carbon beam irradiation at 75% *Dq*. These results imply that, for the induction of SVs, proton beams may have more similar characteristics with other accelerated particles with higher LET than gamma-rays with LET, which is more similar to that of proton beams. *In vitro* analyses by Leloup et al. (2005) and Hada and Sutherland (2006) showed that proton beam irradiation induced much higher numbers of DNA breaks than gamma-ray irradiation, even when the LET of each radiation was adjusted to be similar. Calugaru et al. (2011) also showed that proton beam irradiation always induced a higher number of clustered DNA lesions than gamma-ray irradiation, regardless of the energy of protons. In a track structure analysis, the protons deposited energy in an almost straight cylinder inducing energetic secondary electrons, whereas photon beams with the same LET produced Compton electrons that formed the scattered multidirectional stochastic tracks (Girdhani et al., 2013). This track structure of proton beams may possibly be related to their biological effects in SV induction. Taken together, protons, as accelerated particles with low-LET, have unique features in their mutagenic effects on plant DNA that are distinguished from those of heavy-ion beams with the higher LET and gamma-rays.

### Effect of Irradiation Dose on Mutation Frequency

**FIGURE 9:**
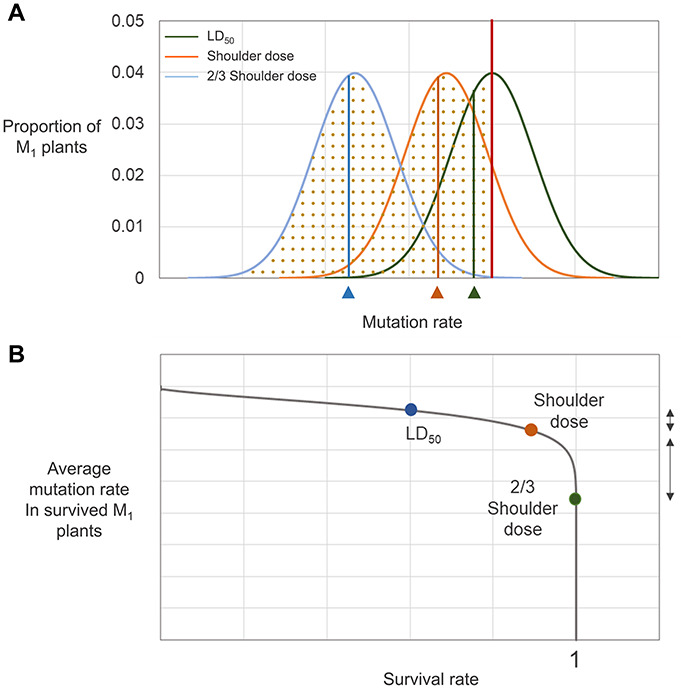
Hypothesized relationship between survival rate and mutation rate. **(A)** Distribution of irradiated individuals with different mutation rates. The red vertical line represents the threshold mutation rate that a survived M_1_ individual can contain. Green, orange, and blue curves are the probability distribution curves for M_1_ plants irradiated at LD_50_, shoulder dose, and two-thirds of shoulder dose, respectively. Dotted regions below each graph indicate the survival rate of individuals irradiated at each dose. The vertical lines on each graph indicates the average mutation rate of the survived individuals for each irradiation treatment. **(B)** Relationship between survival rate and mutation rate derived from **(A)**. The dots represent survival rate and average mutation rate of survived plants determined from **(A)**. The direction arrows on the right side of the graph indicate differences in mutation rate between LD_50_ and shoulder dose-irradiated individuals (shorter arrow) and between shoulder dose and two-thirds of shoulder dose-irradiated individuals (longer arrow).

Although the relationship between irradiation dose and frequency of phenotypic mutations in M_2_ generations has been analyzed for a wide range of doses (Yamaguchi et al., 2009; Kazama et al., 2011; Kazama et al., 2017), comparisons of DNA mutation frequency among plant samples irradiated at different doses have been reported for only a limited range of doses below the shoulder dose (Hase et al., 2018, 2020). In our whole genome sequencing analysis of plant groups irradiated with proton beams at doses near the LD_50_, *Dq*, and two-thirds *Dq*, differences in the frequencies of SBSs, small InDels, and SVs were much smaller among groups for LD_50_ and *Dq* compared with those among groups for *Dq* and two-thirds *Dq* (Figure 8). This result indicates that to increase dose in the range higher than the *Dq* was much less effective than to increase dose in the range below the *Dq* in increasing mutation frequency. Phenotypic mutation frequencies reported previously are consistent with our result. For example, the highest number of rice M_2_ mutant lines per irradiated M_1_ seeds was detected for gamma-ray and ion beam irradiations at the shoulder dose, which indicates that the extent of survival rate decrease in the M_1_ population was not proportional to the increase in mutation frequency in the M_2_ population when the irradiation dose was higher than the shoulder dose (Yamaguchi et al., 2009). Irradiation doses above a certain level greatly decreased the flowering rate of M_1_ Arabidopsis plants; however, argon and iron beam irradiation doses above the same level did not increase the rate of albino plants in the M_2_ population (Kazama et al., 2008). On the basis of these accumulated data, we hypothesized that there may be a threshold mutation rate that an individual plant survived after irradiation with a specific radiation can contain. Assuming that individuals with various rates of mutation are generated along a probability distribution curve by irradiation at specific dose, then only the individuals with mutation rates below a threshold will survive (Figure 9A). Therefore, increasing irradiation dose may not increase mutation rate to be higher than the thresholds, but it may increase the average mutation rate in surviving individuals to a level that is closer to the threshold mutation rate. According to this hypothesis, the distribution curve for plants irradiated at the shoulder dose will have the smallest slope at the threshold mutation rate. Because of this characteristic, the survival rates at doses higher than the shoulder dose drop sharply, but the average mutation rate among the individuals that survived does not increase that much (Figure 9B). In contrast, for doses below the shoulder dose, the ratio of mutation rate increase to survival rate decrease is much larger. This relationship between survival rate and mutation rate in our “threshold mutation rate hypothesis” fits well with the results of the present and previous studies. Further whole genome sequencing analysis of plant samples irradiated at various doses may provide more information to determine threshold mutation rates and the exact distribution pattern of irradiated samples according to the mutation rate.

### Characteristics of Radiation-Induced DNA Breaks and Repair

We analyzed SVs induced from proton beam and gamma-ray irradiation by examining DNA rearrangement junctions at the nucleotide level. Most of the SVs had simple structures and were expected to be derived from a single DNA rearrangement event, unlike previously analyzed SVs that were induced by heavy ion beams and were mostly complex involving a series of successive rearrangements (Hirano et al., 2015; Kazama et al., 2017). The structural simplicity of the SVs in our study enabled the determination of SV types and separate analysis for each rearrangement events. Most of the DNA rearrangement events (34 of 41) involved joining of end sequences with microhomology (2–23 bp). Consistent with our results, the involvement of microhomologous sequences in DNA structural rearrangements has been reported previously in plant mutant lines developed by irradiation (Shirley et al., 1992; Shikazono et al., 2001; Kazama et al., 2017). DSBs and DNA repair by the error-prone NHEJ has been suggested as the mechanism for induction of SVs by irradiation in plants (Hirano et al., 2015). The presence or absence of microhomologous sequences and InDels around microhomologous sequences is an important factor for subclassification of the NHEJ mechanism (Mcvey and Lee, 2008). Because conservation or the presence of small InDels adjacent to a DSB can be examined in the inversion events that accounted for the largest portion of the DNA rearrangements in our study, we analyzed the rearrangement junctions in inversion events to characterize the NHEJ mechanism. Among the 23 inversions, 12 were expected to be associated with end joining via microhomologous sequences in the original DNA molecule and did not contain any deletions around DSB sites, although they did contain small deletions on the joined microhomologous sequences. This finding implies that DSBs and ligation occurred on or at the ends of microhomologous sequences in those events, which excluded the possibility that an alternative mechanism of NHEJ, microhomology-mediated end joining (MMEJ), was involved. In MMEJ the DNA regions between break points and microhomologous sequences are removed for end joining using microhomology (Ottaviani et al., 2014). The preference of using microhomology in NHEJ might be a reason for frequent presence of microhomologous sequences on the rearrangement junctions. Although microhomology is not essential for NHEJ, the use of microhomology may be dominant in the joining of DNA ends if there are microhomologous sequences in the overhangs (Gerstein and Lieber, 1993; Weinstock et al., 2007; Pannunzio et al., 2014). In yeasts, which have NHEJ systems that show high similarity to those of plants in its components, there is more reliance on terminal microhomology in NHEJ than there is in mammals (Daley and Wilson, 2005; Daley et al., 2005; Lieber, 2010). Therefore, coincidental DSBs near or at the ends of two microhomologous sequences by proton beam irradiation may lead to SV events using microhomology rather than re-joining to the original DNA without using microhomology. Besides these inversions, inversion events with very small InDels around rearrangement junctions (<10 bp; 6 of 23 inversion events) are likely to be mediated by classical NHEJ (cNHEJ). This is because deletion of a few nucleotides is often associated with cNHEJ due to the iterative and unordered actions of nuclease, polymerases, and ligase in this process (Lieber, 2010). Deletions longer than 10 bp around microhomologous sequences were found in four inversion events. MMEJ could be a mechanism for these SV. In the Arabidopsis mutant lacking KU70 (an essential component of cNHEJ), inversions induced using the CRISPR/Cas system resulted in a high proportion of rearrangement junctions joined by microhomologous sequences with deletions (10 s to > 100 nucleotides), which indicates that MMEJ can occur in plants as an alternative to cNHEJ (Schmidt et al., 2019). Therefore, we suggest that cNHEJ was responsible for most of the DNA rearrangements found in the proton beam-irradiated Arabidopsis, and MMEJ may be involved as a minor mechanism. Hirano et al. (2015) speculated that alternative NHEJ, rather than cNHEJ, might contribute to a high rate of SVs because there were frequent deletions at rearrangement junctions which were joined via microhomologous sequences in argon- and ion beam-irradiated Arabidopsis. Hase et al. (2012) showed that the sensitivity of Arabidopsis to radiation was higher in a DNA ligase IV mutant lacking the cNHEJ pathway when it was compared to wild-type Arabidopsis. The degree of the survival rate decrease according to increase of irradiation dose was much higher in low-LET carbon beam irradiation (113 keV μm^–1^) than it was in high-LET carbon beam irradiation (425 keV μm^–1^), which implies that the contribution of cNHEJ was higher in the repair of DNA damage by low-LET radiation than it was for high-LET radiation. The higher contribution of cNHEJ in SV generation found in irradiation of proton beams with low LET in our study may support the relationship between LET and the DNA repair mechanism reported previously.

## Conclusion

Irradiation of proton beams and gamma-rays at various irradiation doses showed that the survival rate in M_1_ generation and mutation rate in M_2_ generation were dependent on irradiation doses. At the irradiation doses that had similar impacts on survival rate, the Arabidopsis M_2_ lines derived from proton beam-irradiation contained more SVs compared to those from gamma-irradiation. However, the frequency of small mutations including SBSs and small InDels was similar between two irradiated groups. The SVs were expected to be induced by NHEJ involving microhomologous end sequences, and to be related to high frequency and the broad spectrum of phenotypic mutations in M_2_ lines derived from proton beam-irradiation. Comparative analysis with heavy ion beams showed that proton beams induced more frequent small mutations than heavy ion beams. Therefore, protons, as radioactive particles with low-LET, have unique characteristics in mutagenesis of plant DNA that are distinguished from those of gamma-rays and heavy-ion beams. These finding are expected to be the basis for plant mutation breeding and functional genomics studies using proton beams.

## Data Availability Statement

The raw sequence datasets produced for this study were deposited to the Sequence Read Archive (SRA) database (www.ncbi.nlm.nih.gov/sra) of National Center for Biotechnology Information (BioProject ID: PRJNA751183).

## Author Contributions

SL participated in population generation, phenotype analysis, sequence data analysis, and manuscript writing. Y-JK participated in validation of the SVs. IB participated in the sequence analysis. H-IC reviewed the manuscript and participated in the discussion. J-WA participated in the phenotype analysis. J-BK, S-YK, and SK managed the project that supported this research. YJ participated in the design of the study, sequence data analysis, SV validation, and manuscript writing. All authors contributed to the article and approved the submitted version.

## Conflict of Interest

The authors declare that the research was conducted in the absence of any commercial or financial relationships that could be construed as a potential conflict of interest.

## Publisher’s Note

All claims expressed in this article are solely those of the authors and do not necessarily represent those of their affiliated organizations, or those of the publisher, the editors and the reviewers. Any product that may be evaluated in this article, or claim that may be made by its manufacturer, is not guaranteed or endorsed by the publisher.

## Footnotes

1
http://broadinstitute.github.io/picard/
