# Supplementary material for: Mutagenic Effect of Proton Beams Characterized by Phenotypic Analysis and Whole Genome Sequencing in Arabidopsis
Source: Front Plant Sci. 2021 Oct 28;12:752108. doi: 10.3389/fpls.2021.752108 (PMC8581144; doi:10.3389/fpls.2021.752108)
Supplement: Supplementary file 1 [file Table_1.DOCX]

Supplementary Table S1. Information on next-generation sequencing read production and mapping for Arabidopsis wild-type and M_2_ lines derived from proton beam- and gamma-irradiation

| Sample name | Raw sequencing data | | Trimmed sequencing data | | | Number of reads mapped on reference genome^Z^ (%) | Mapped region on reference genome (%) |
| --- | --- | --- | --- | --- | --- | --- | --- |
|  | Number of reads | Total length (bp) | Number of reads | Total length (bp) | Genome  coverage |  |  |
| Non-irradiated-1 | 78,435,654 | 7,921,891,974 | 65,808,804 | 5,830,797,700 | 43.19X | 52,266,555 (79.42%) | 116,249,626 (97.78%) |
| Non-irradiated-2 | 90,327,196 | 9,123,046,796 | 76,341,674 | 6,885,571,720 | 51.00X | 60,906,850 (79.78%) | 116,255,934 (97.78%) |
| G900-1 | 76,046,956 | 7,680,742,556 | 62,794,704 | 5,430,967,810 | 40.23X | 47,256,130 (75.25%) | 116,249,271 (97.78%) |
| G900-2 | 85,704,590 | 8,656,163,590 | 70,920,496 | 6,197,006,754 | 45.91X | 52,846,438 (74.52%) | 116,250,928 (97.78%) |
| G900-3 | 89,125,792 | 9,001,704,992 | 74,498,996 | 6,686,705,774 | 49.53X | 56,234,602 (75.48%) | 116,251,122 (97.78%) |
| G900-4 | 86,757,702 | 8,762,527,902 | 71,323,908 | 6,160,345,178 | 45.63X | 53,509,821 (75.02%) | 116,252,783 (97.78%) |
| G900-5 | 70,816,778 | 10,693,333,478 | 62,294,404 | 7,743,381,211 | 57.36X | 47,561,606 (76.35%) | 116,239,201 (97.77%) |
| G900-6 | 71,329,244 | 10,770,715,844 | 62,266,862 | 7,691,302,903 | 56.97X | 42,885,050 (68.87%) | 116,237,066 (97.77%) |
| P494-1 | 78,395,734 | 7,917,969,134 | 66,340,544 | 5,930,689,128 | 43.93X | 20,881,847 (76.70%) | 116,231,055 (97.76%) |
| P494-2 | 89,254,710 | 9,014,725,710 | 75,987,612 | 6,685,522,058 | 51.00X | 57,415,315 (75.56%) | 116,252,159 (97.78%) |
| P494-3 | 79,264,536 | 8,005,718,136 | 65,473,630 | 5,769,707,128 | 42.74X | 50,163,528 (76.62%) | 116,249,616 (97.78%) |
| P494-4 | 77,717,702 | 7,849,487,902 | 63,231,956 | 5,566,122,044 | 41.23X | 47,004,535 (74.34%) | 116,247,267 (97.78%) |
| P494-5 | 64,634,650 | 9,759,832,150 | 55,340,350 | 6,692,278,211 | 49.57X | 41,354,175 (74.73%) | 116,236,993 (97.77%) |
| P494-6 | 60,716,286 | 9,168,159,186 | 53,065,712 | 6,507,925,843 | 48.21X | 39,467,383 (74.37%) | 116,233,417 (97.76%) |
| P787-1 | 75,780,954 | 7,653,876,354 | 62,533,504 | 5,521,764,238 | 40.90X | 49,486,849 (79.14%) | 116,247,063 (97.78%) |
| P787-2 | 78,850,806 | 7,963,931,406 | 65,581,670 | 5,849,098,938 | 43.33X | 52,373,011 (79.86%) | 116,248,503 (97.78%) |
| P787-3 | 79,219,206 | 8,001,139,806 | 65,852,412 | 5,861,232,208 | 43.42X | 50,868,517 (77.25%) | 116,247,980 (97.78%) |
| P787-4 | 93,929,036 | 9,486,832,636 | 76,215,180 | 6,594,302,390 | 48.84X | 63,468,214 (83.28%) | 116,250,287 (97.79%) |
| P787-5 | 64,526,758 | 9,743,540,458 | 56,190,302 | 6,889,883,961 | 51.04X | 42,051,082 (74.84%) | 116,236,745 (97.77%) |
| P787-6 | 56,530,776 | 8,536,147,176 | 49,900,086 | 6,197,973,609 | 45.91X | 37,690,068 (75.53%) | 116,228,603 (97.76%) |
| P995-1 | 75,829,690 | 7,658,798,690 | 60,848,490 | 5,217,041,756 | 38.65X | 47,594,328 (78.22%) | 116,247,481 (97.78%) |
| P995-2 | 81,613,776 | 8,242,991,376 | 67,980,924 | 5,892,692,156 | 43.65X | 52,686,628 (77.50%) | 116,252,744 (97.78%) |
| P995-3 | 75,836,044 | 7,659,440,444 | 63,267,358 | 5,521,341,704 | 40.90X | 50,331,576 (79.55%) | 116,250,021 (97.78%) |
| P995-4 | 86,740,056 | 8,760,745,656 | 71,188,658 | 6,270,525,036 | 46.45X | 52,894,363 (74.30%) | 116,228,398 (97.76%) |
| P995-5 | 65,964,048 | 9,960,571,248 | 57,896,742 | 7,160,761,065 | 53.04X | 42,733,934 (73.81%) | 116,239,165 (97.77%) |
| P995-6 | 59,792,380 | 9,028,649,380 | 52,216,316 | 6,450,169,250 | 47.78X | 40,455,984 (77.48%) | 116,235,966 (97.77%) |
| Average | 76,659,272 | 8,731,641,692 | 64,436,973 | 6,277,119,607 | 46.55X | 48,168,784 (74.75%) | 116,244,208 (97.78%) |

^Z^ Reference genome: *Arabidopsis thaliana* var. Landsberg *erecta* (Genbank accession number: GCA_001651475.1)
