# Supplementary material for: Mutagenic Effect of Proton Beams Characterized by Phenotypic Analysis and Whole Genome Sequencing in Arabidopsis
Source: Front Plant Sci. 2021 Oct 28;12:752108. doi: 10.3389/fpls.2021.752108 (PMC8581144; doi:10.3389/fpls.2021.752108)
Supplement: Supplementary file 4 [file Data_Sheet_1.PDF]

Supplementary Figure S1. Schematic diagram showing the results of PCRs performed to validate structural variations

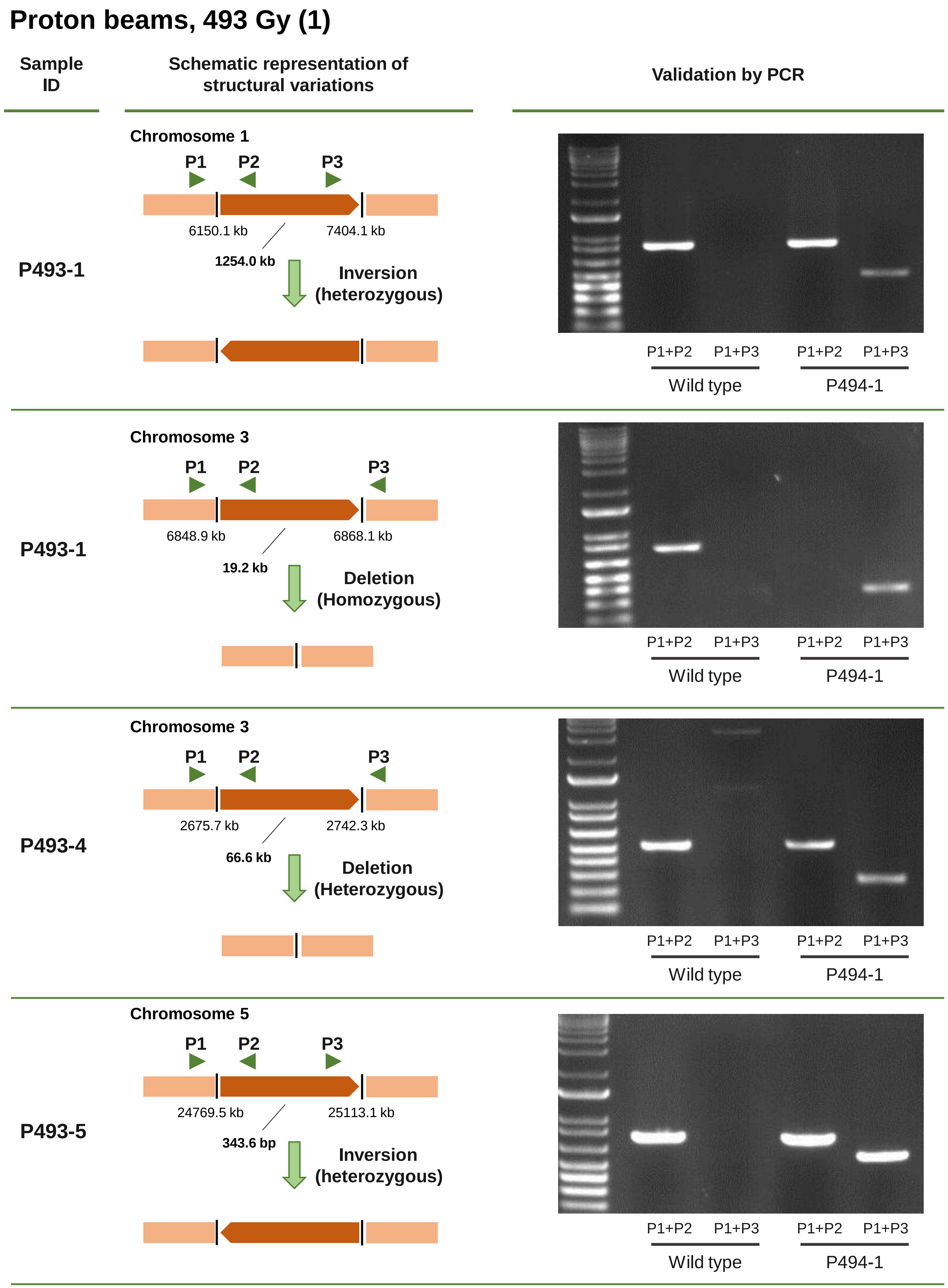

Proton beams, 493 Gy (2)

Sample ID	Schematic representation of structural variations	Validation by PCR
P493-6	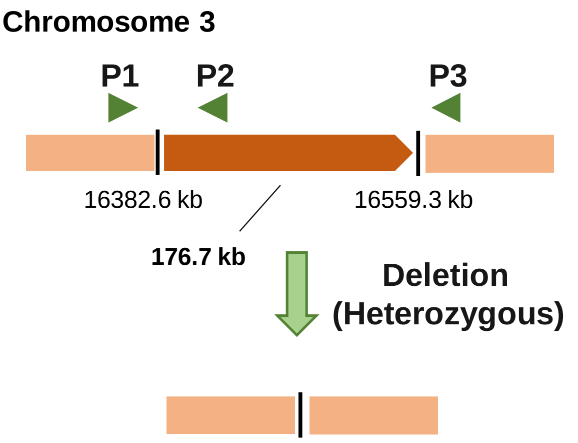 	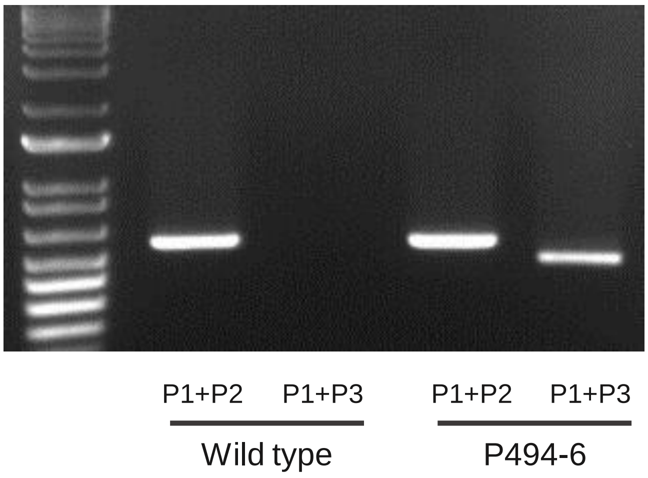

Proton beams, 787 Gy (1)

Sample Number	Schematic representation of structural variations	Validation by PCR
P787-1	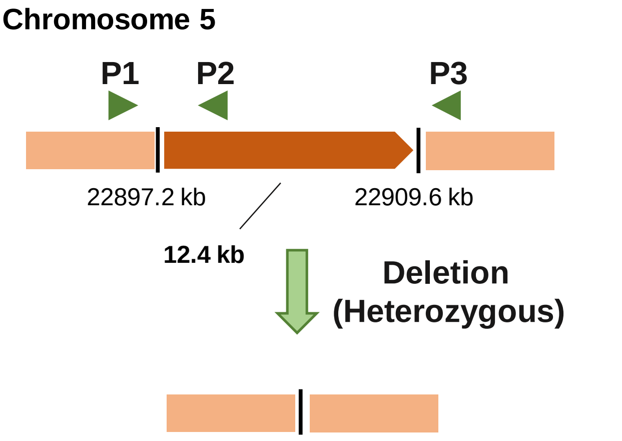	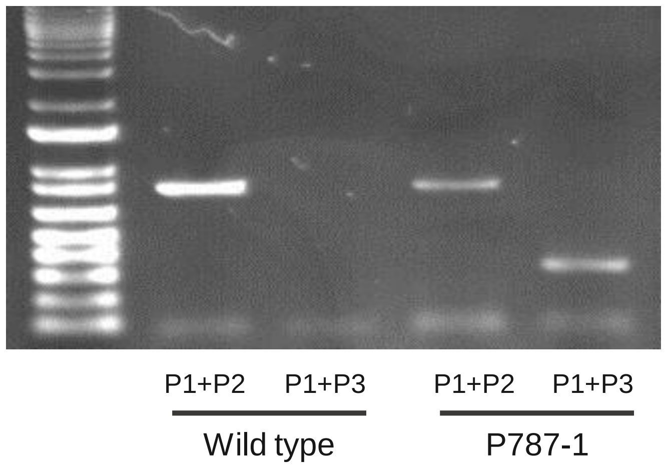
P787-2	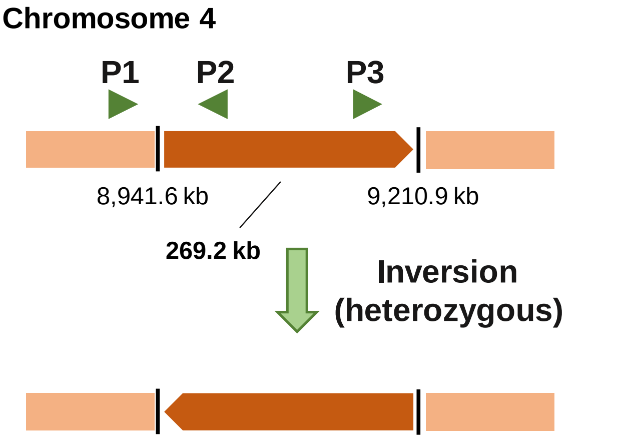	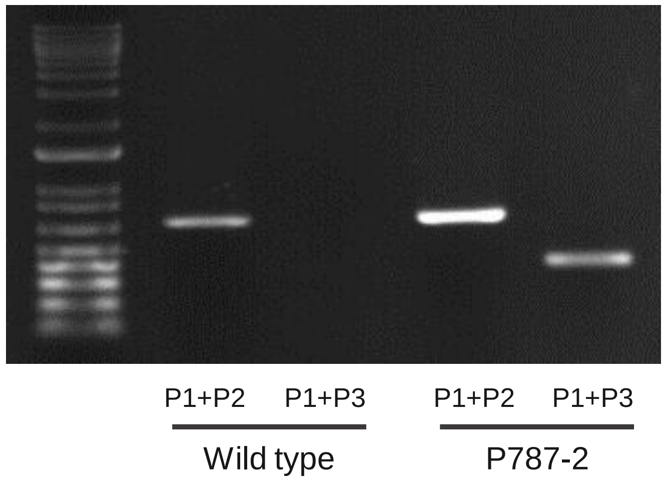
P787-2	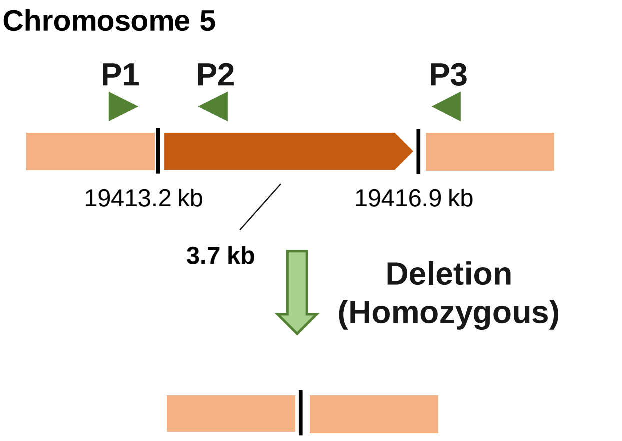	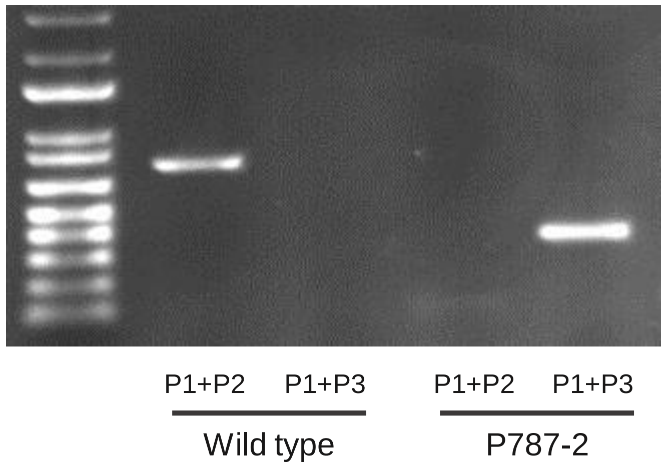
P787-3	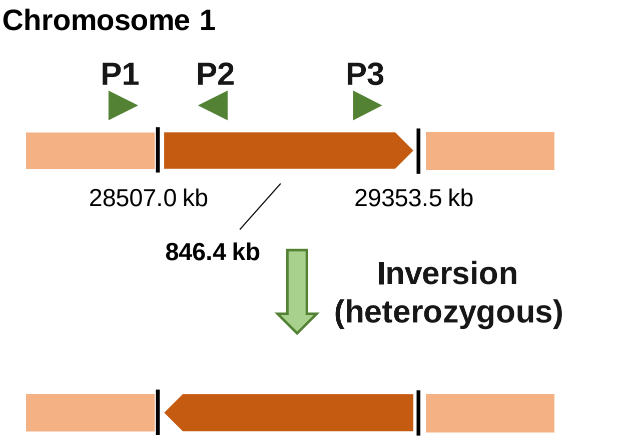	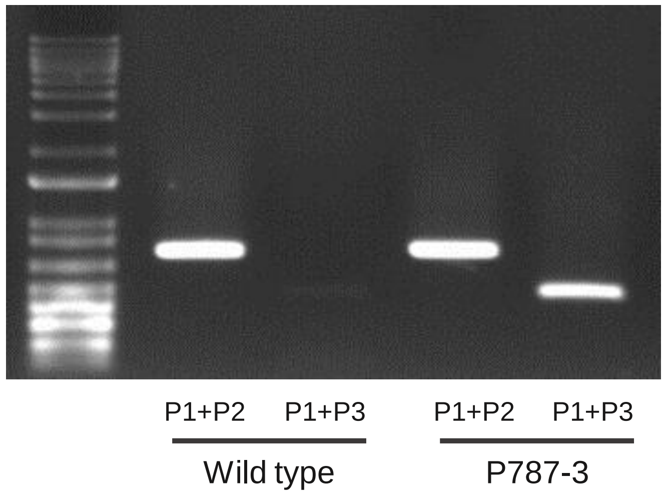

Proton beams, 787 Gy (2)

Sample Number	Schematic representation of structural variations	Validation by PCR

P787-3

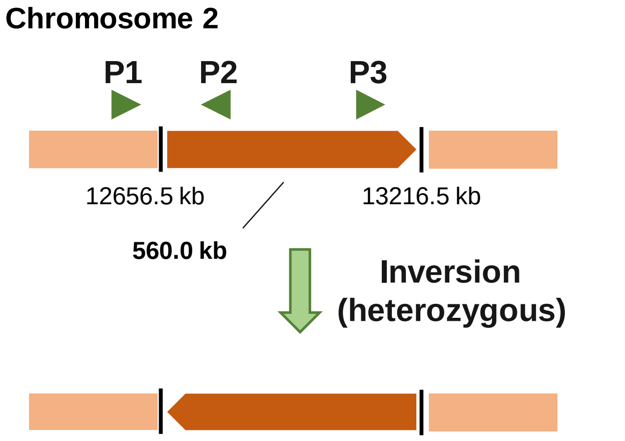

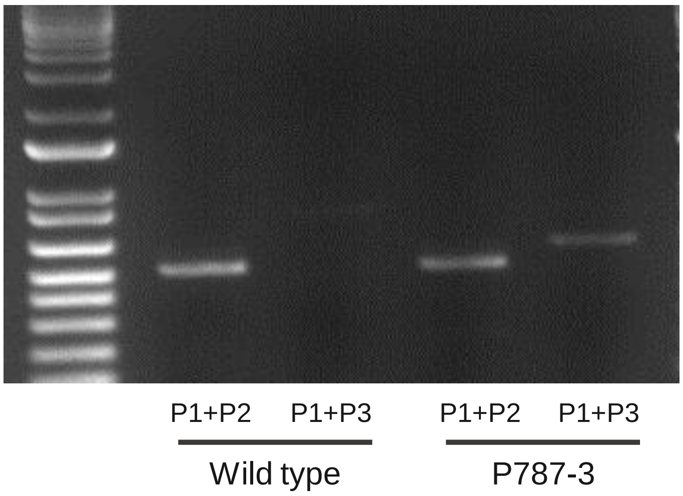

P787-3

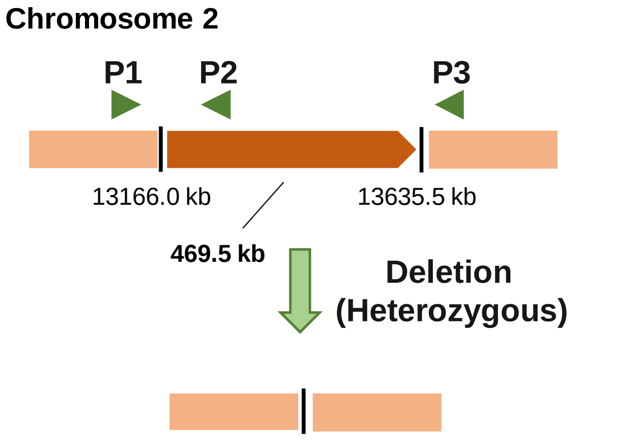

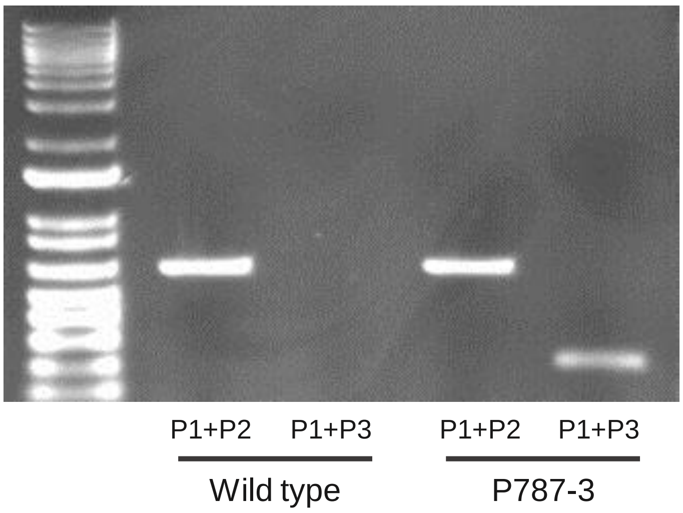

P787-3

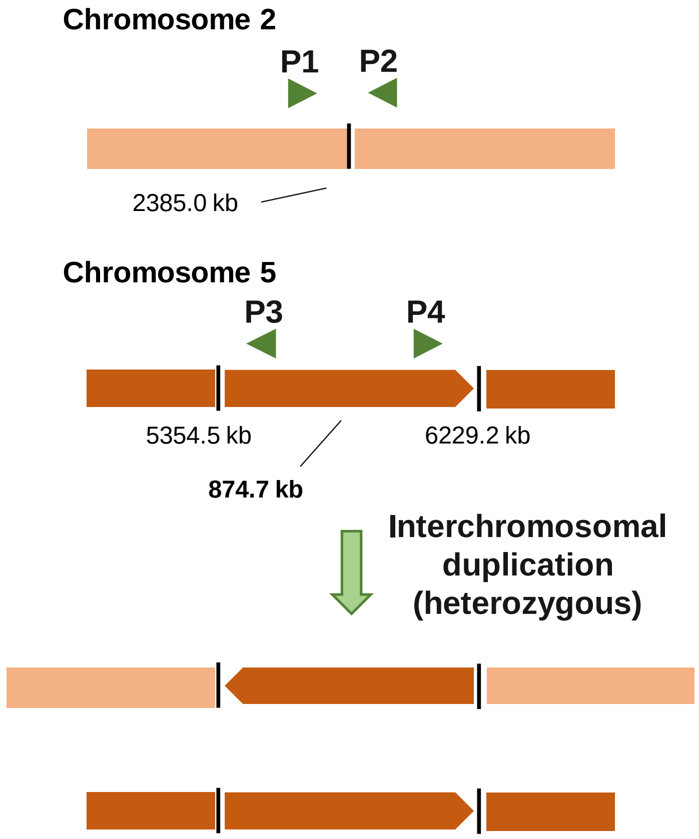

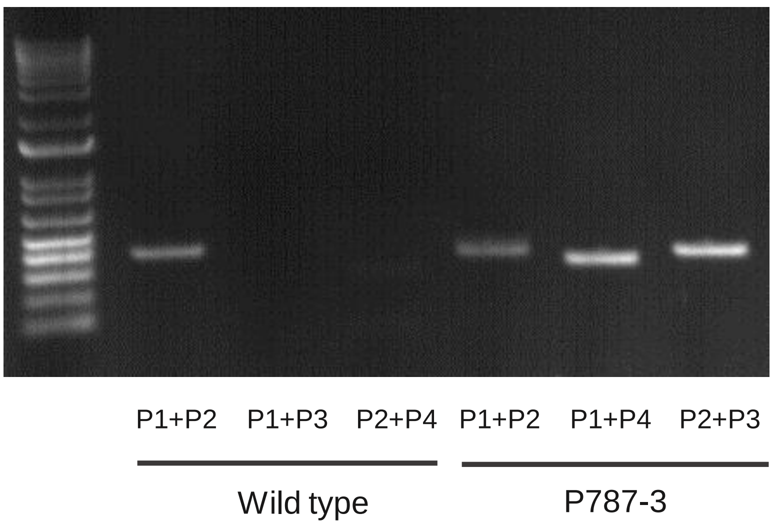

Proton beams, 787 Gy (3)

Sample Number	Schematic representation of structural variations	Validation by PCR

P787-3

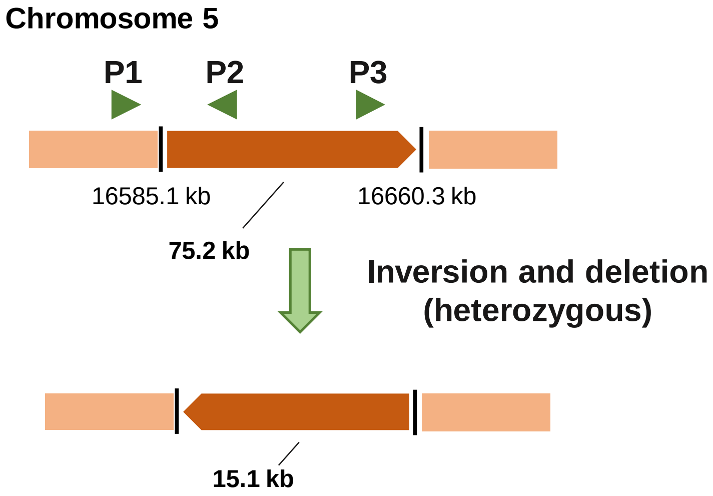

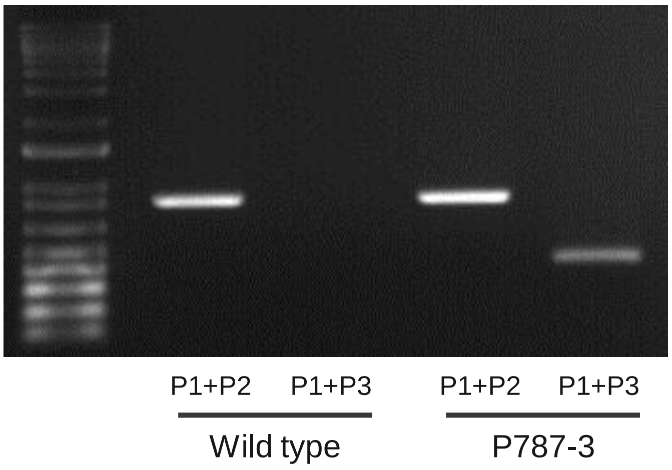

P787-4

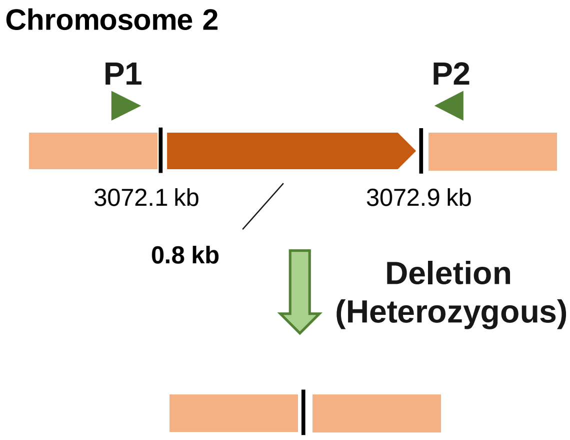

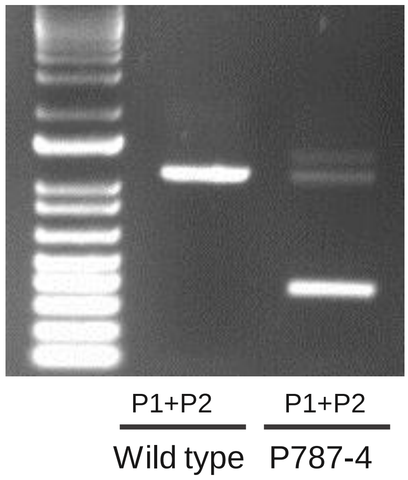

P787-5

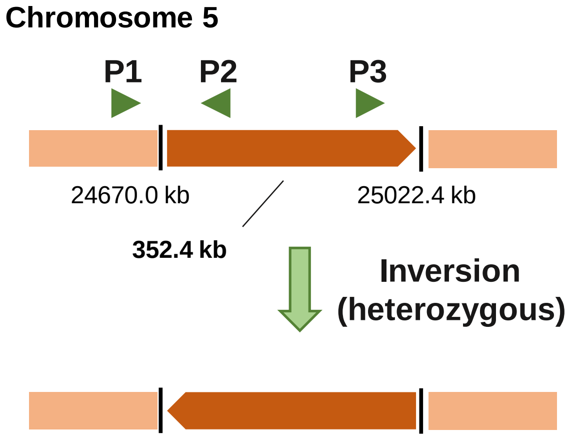

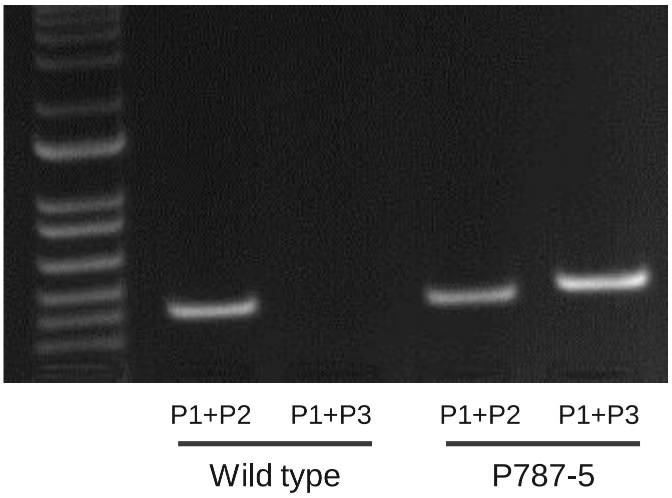

P787-6

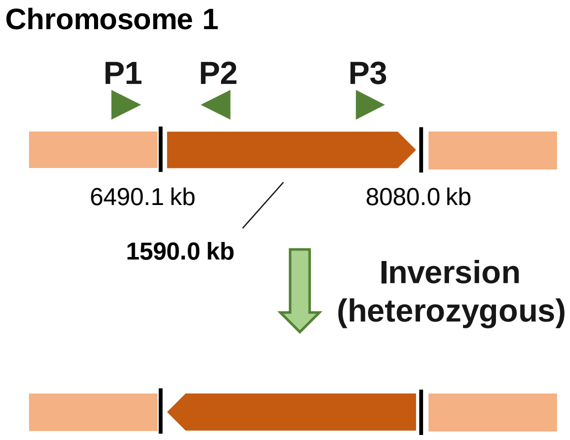

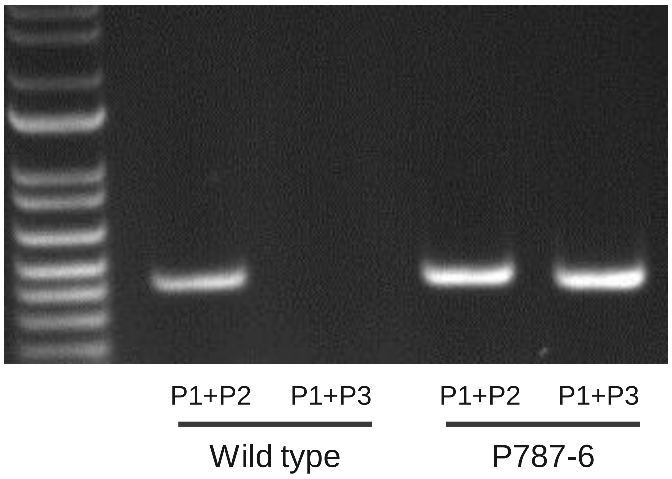

Proton beams, 787 Gy (4)

Sample Number	Schematic representation of structural variations	Validation by PCR

P787-6

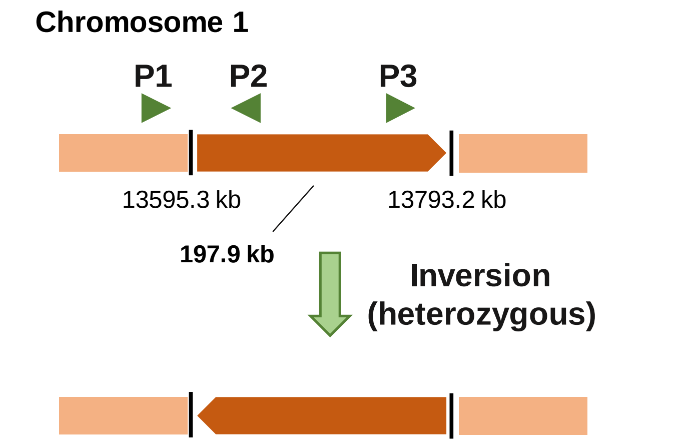

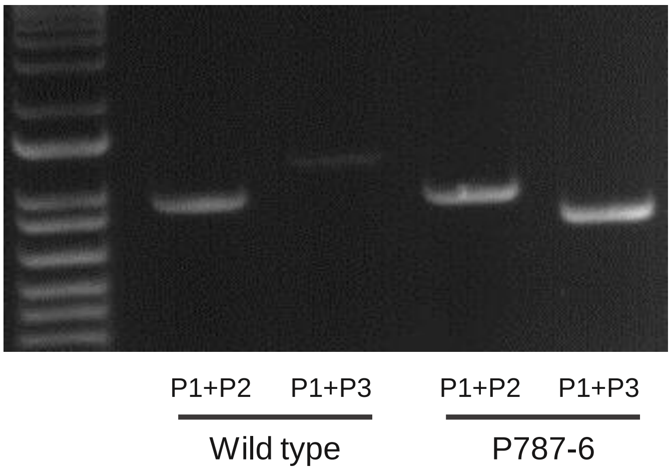

P787-6

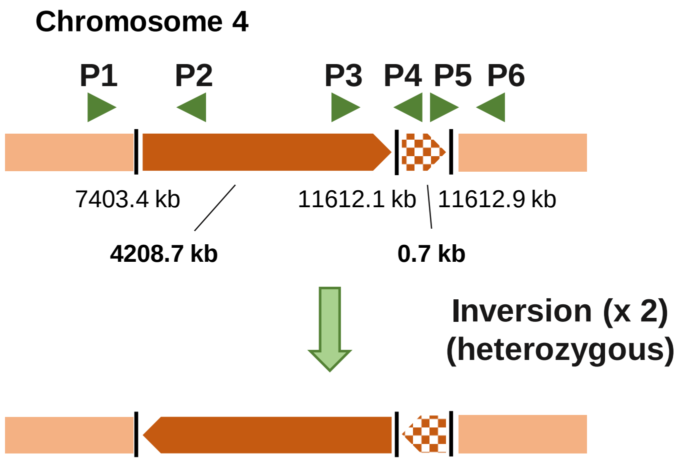

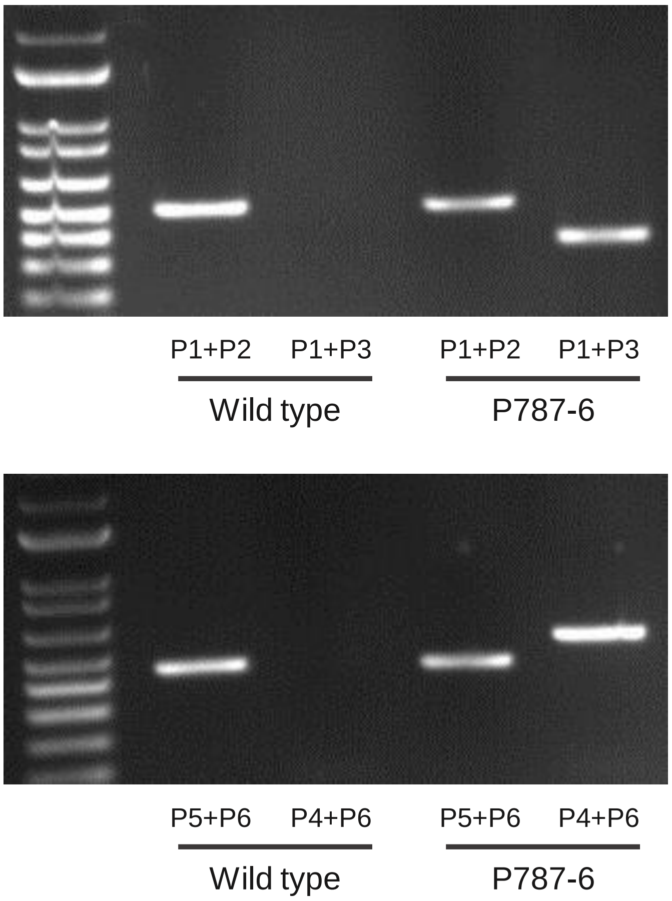

P787-6

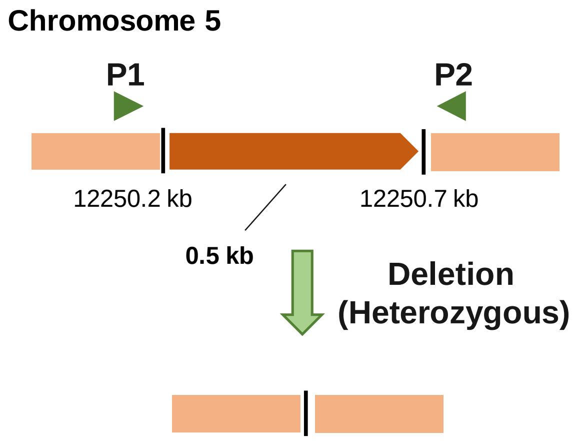

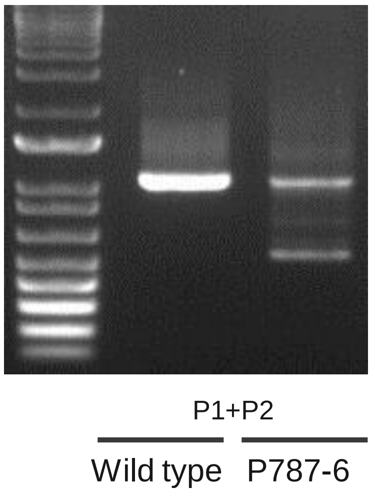

Proton beams, 787 Gy (5)

Sample Number	Schematic representation of structural variations	Validation by PCR
P787-6	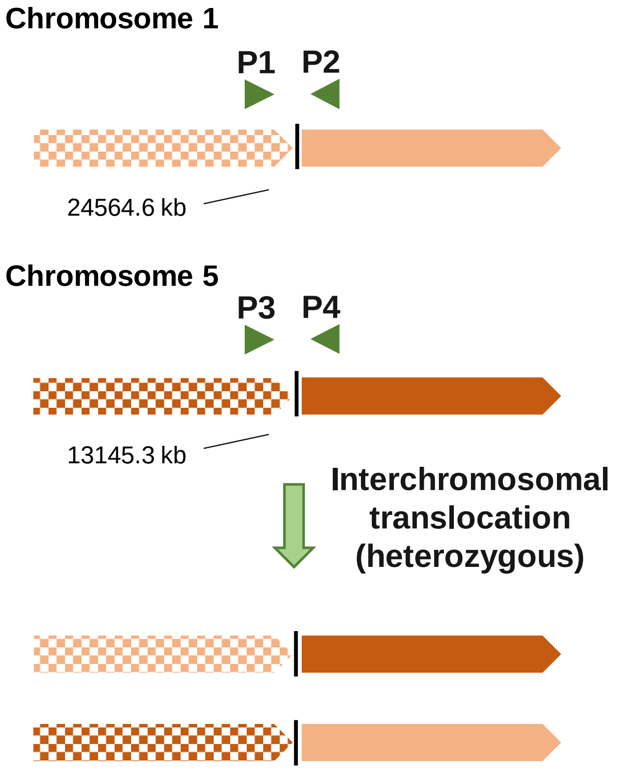	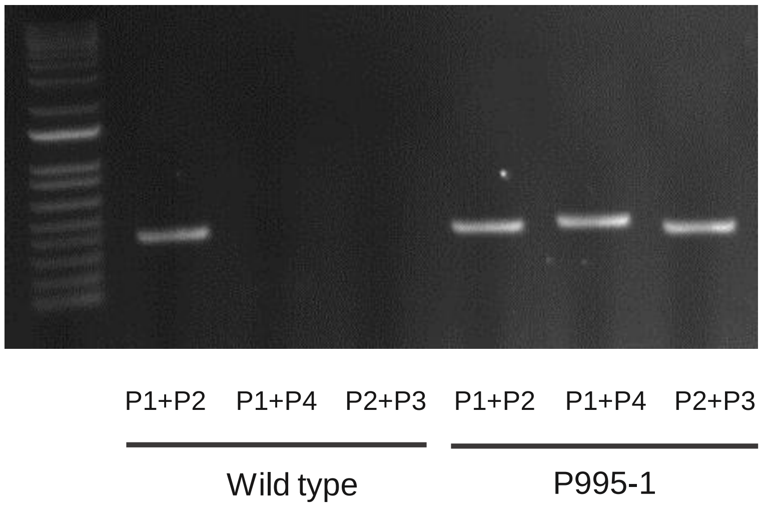

Proton beams, 995 Gy (1)

Sample ID	Schematic representation of structural variations	Validation by PCR
P995-1	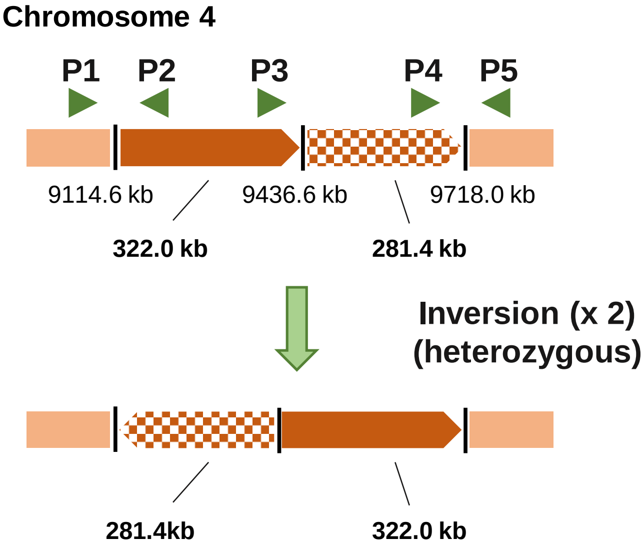	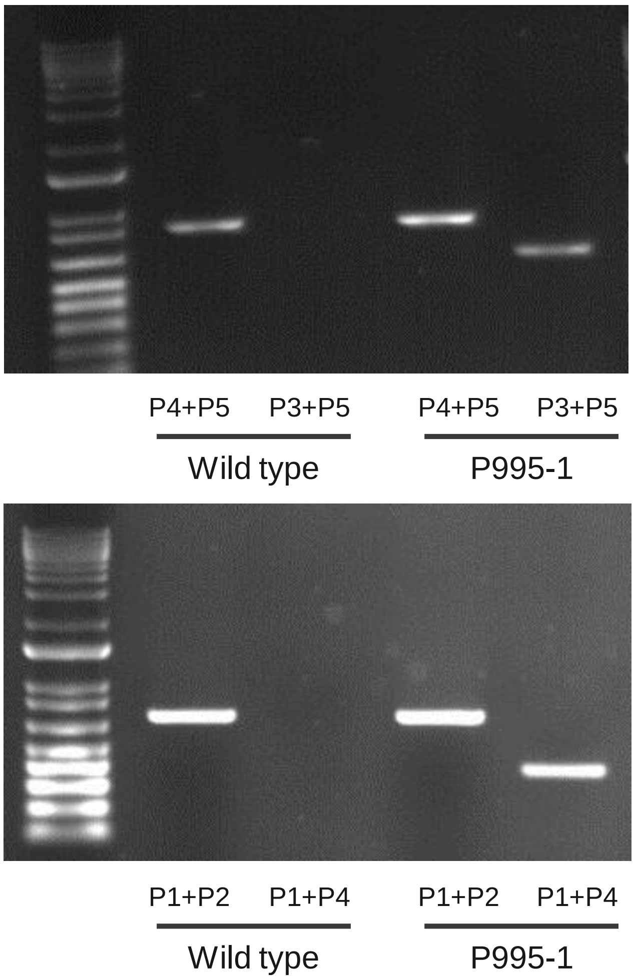
P995-1	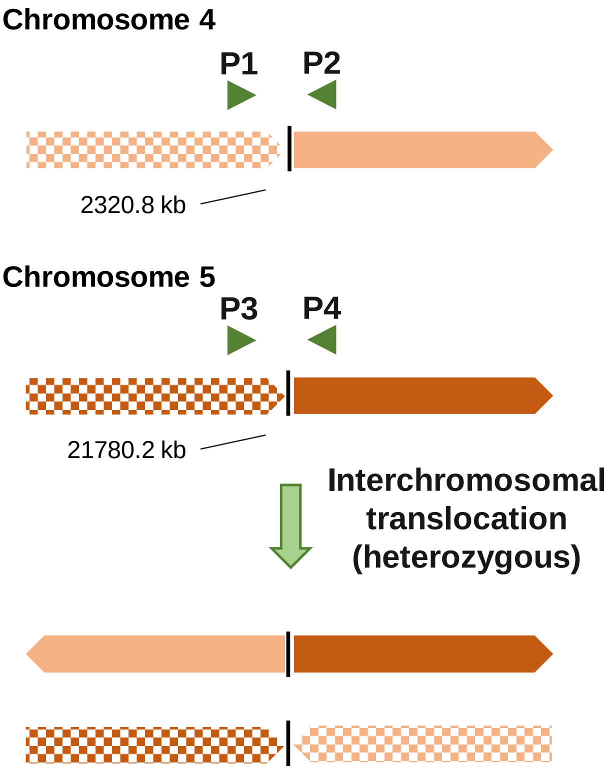	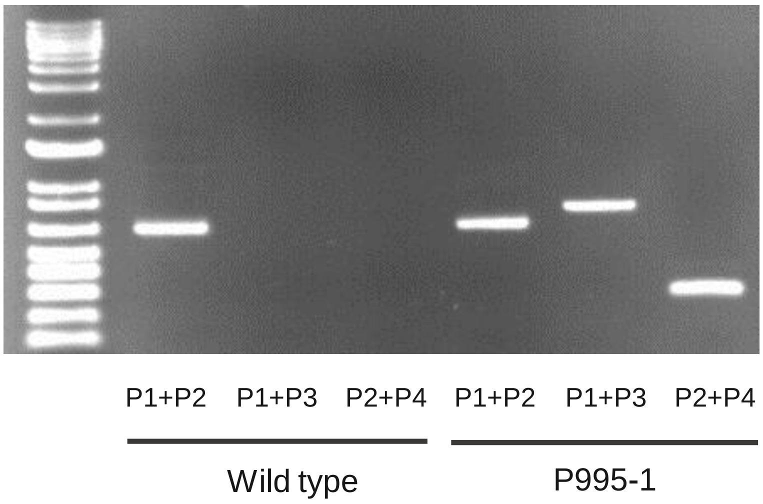

Proton beams, 995 Gy (2)

Sample ID	Schematic representation of structural variations	Validation by PCR

P995-1

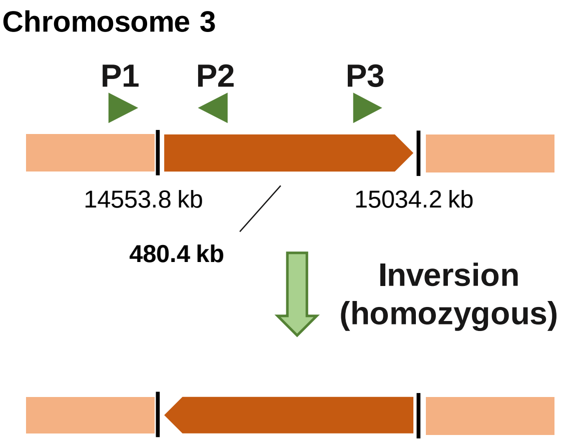

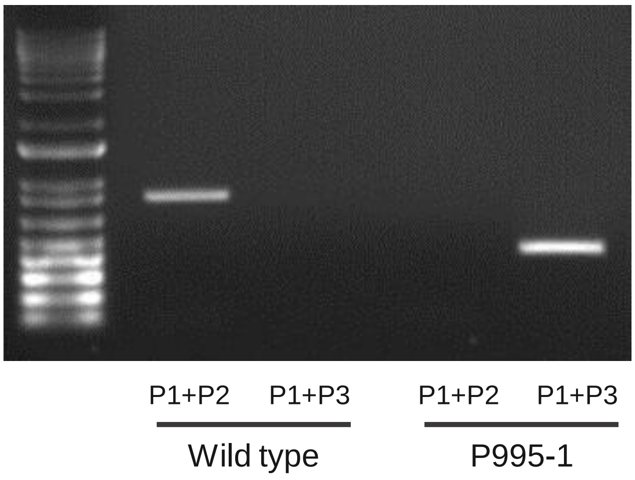

P995-2

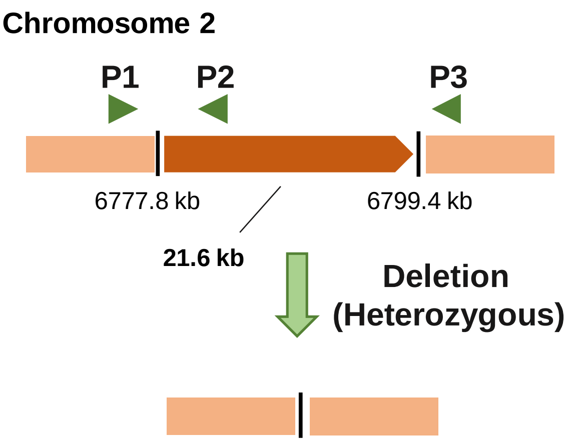

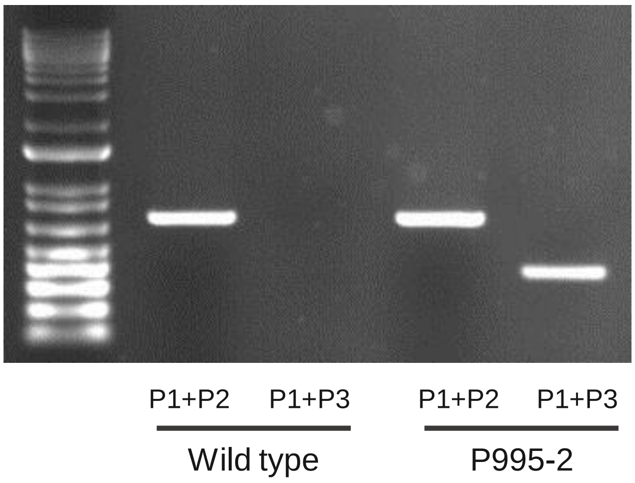

P995-4

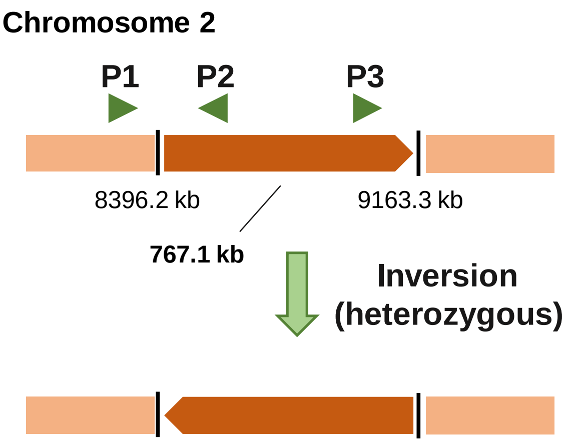

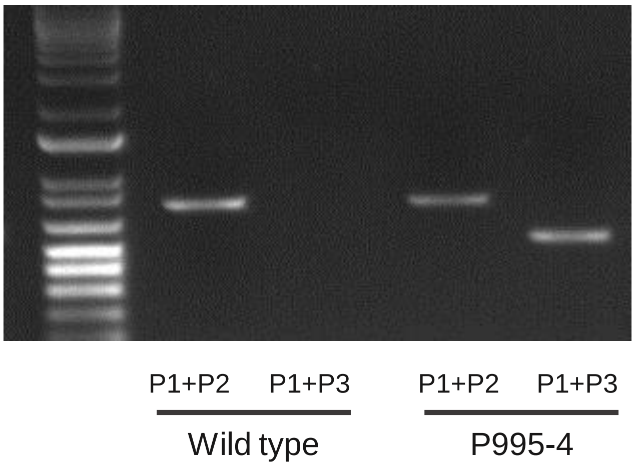

P995-4

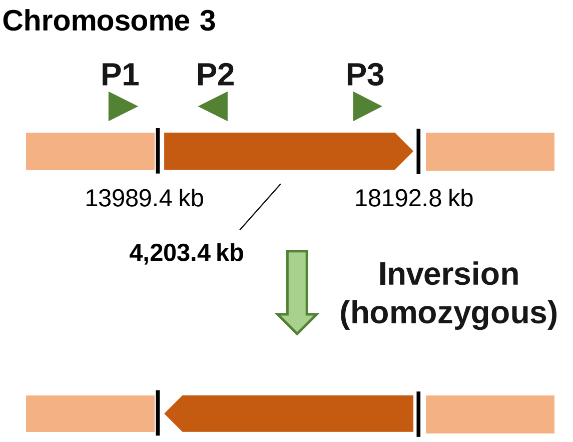

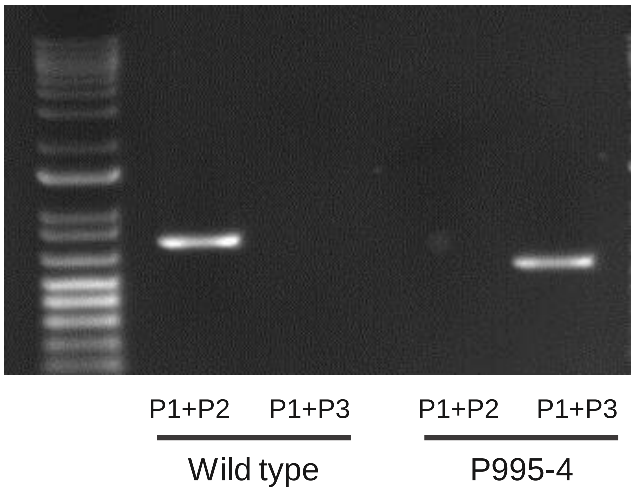

Proton beams, 995 Gy (3)

Sample ID	Schematic representation of structural variations	Validation by PCR

P995-4

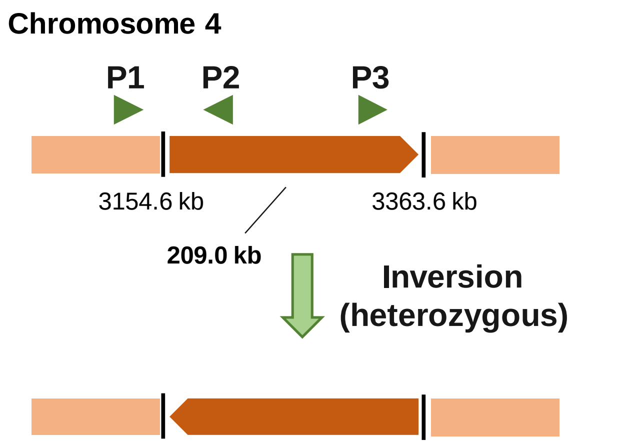

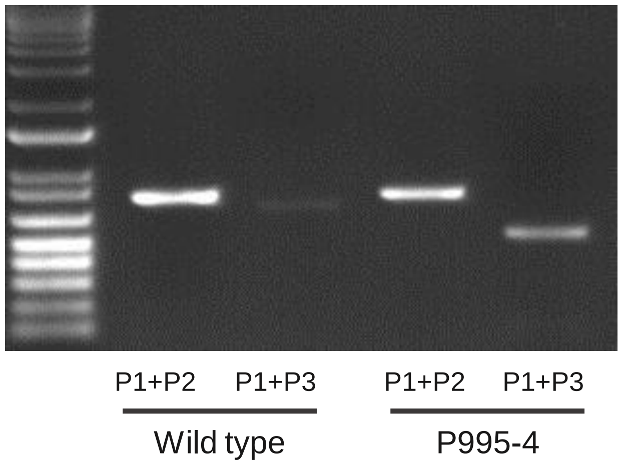

P995-4

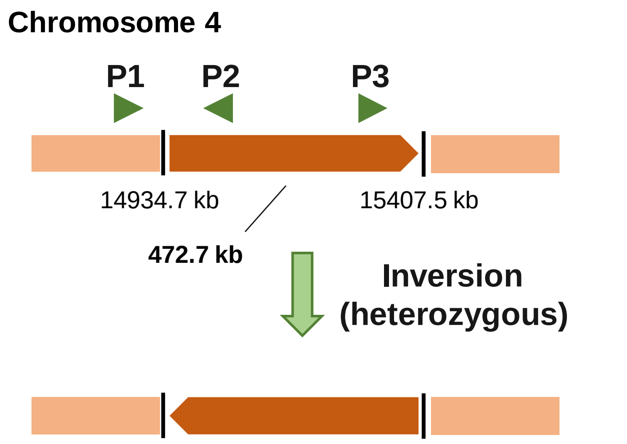

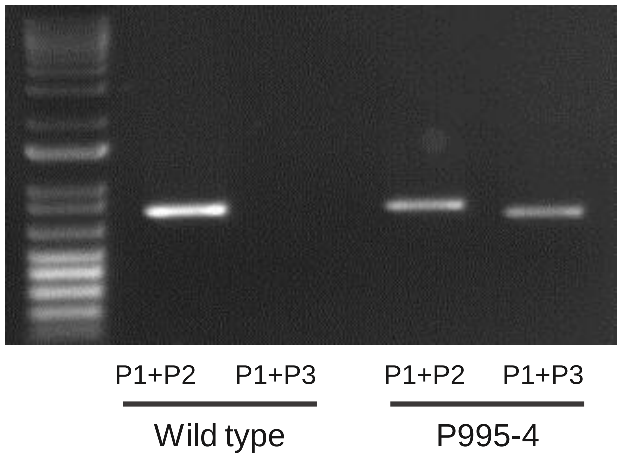

P995-4

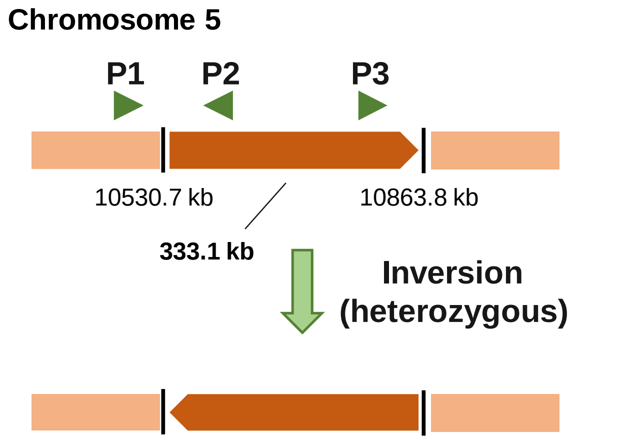

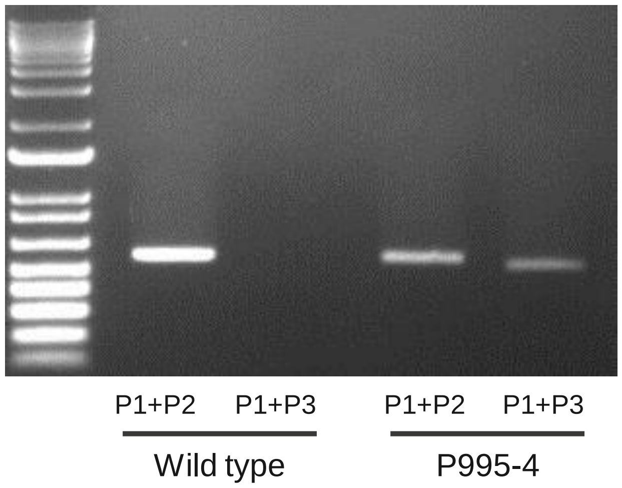

P995-4

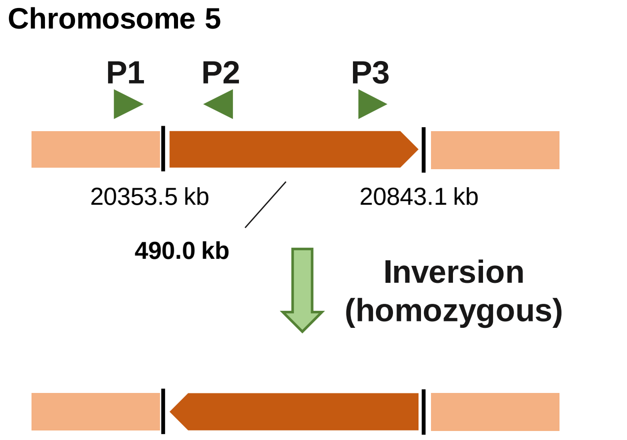

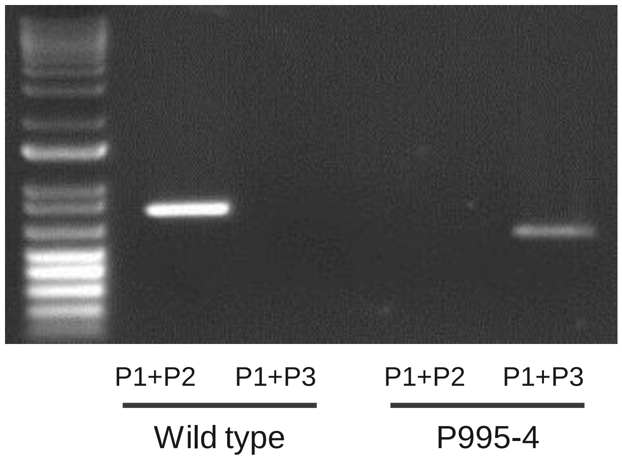

Proton beams, 995 Gy (4)

Sample ID	Schematic representation of structural variations	Validation by PCR
P995-4	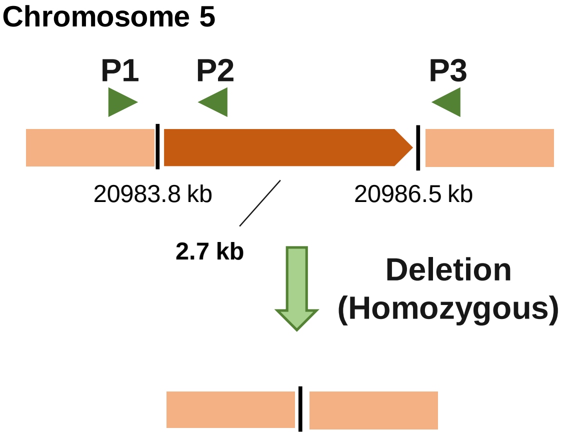	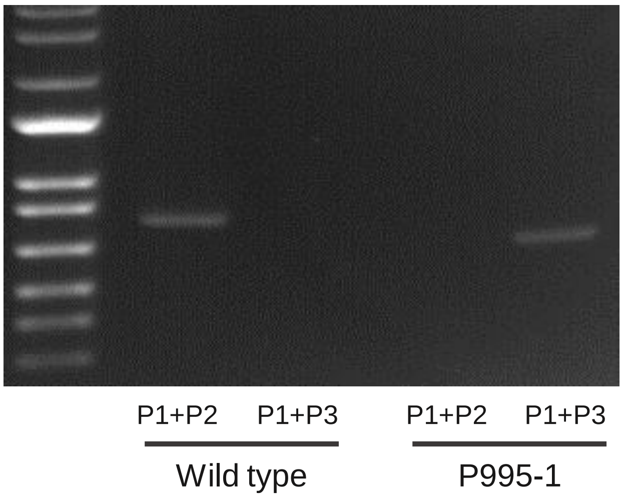
P995-4	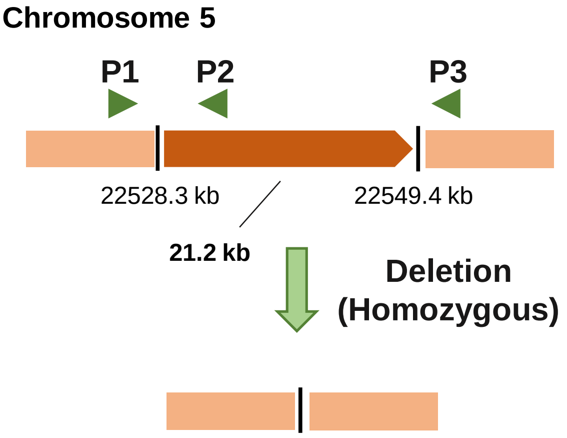	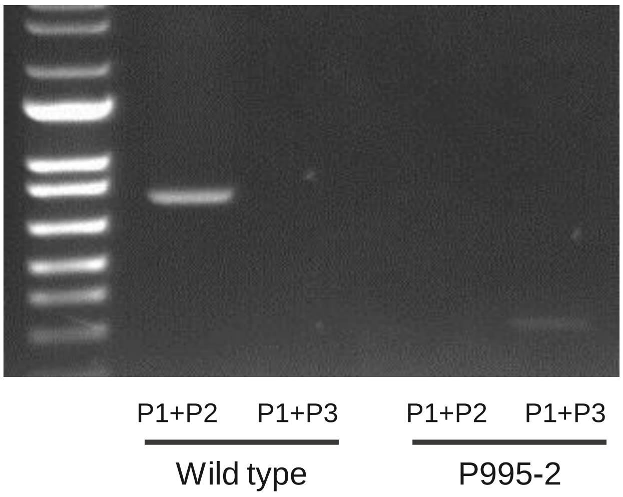
P995-4	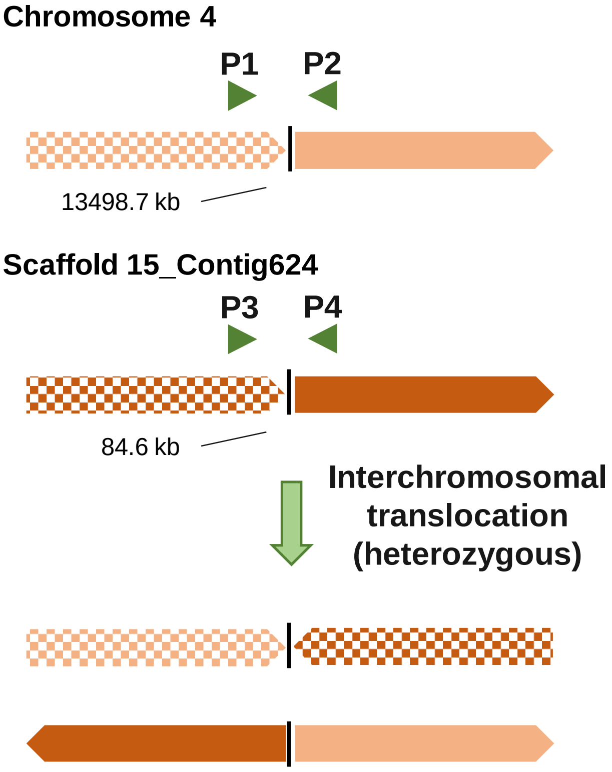	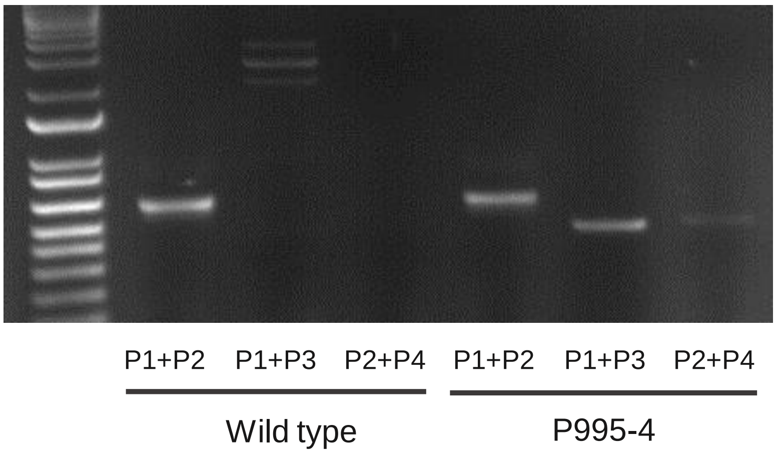

Proton beams, 995 Gy (5)

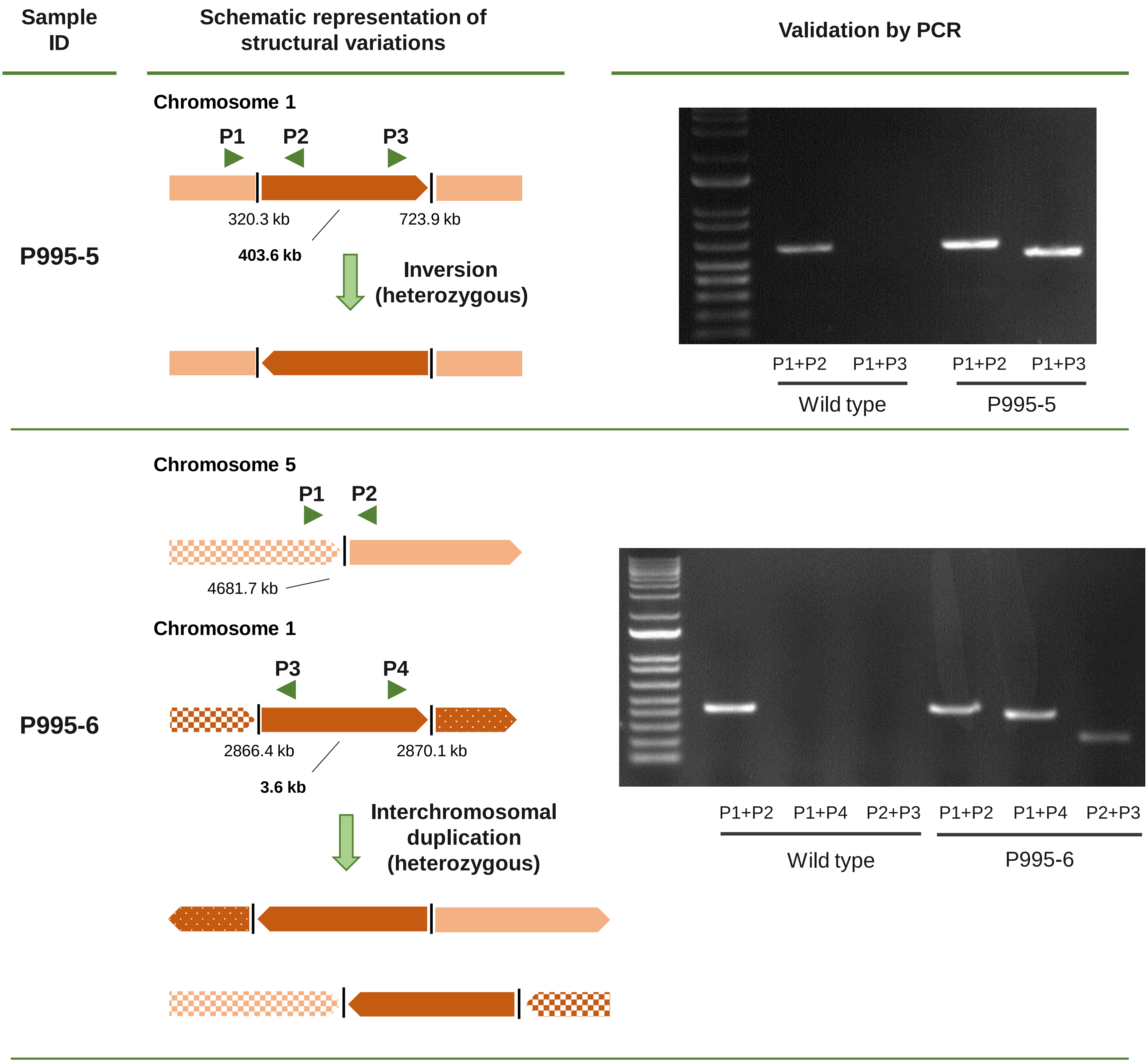

Gamma-rays, 900 Gy (1)

Sample Number	Schematic representation of structural variations	Validation by PCR

G900-1

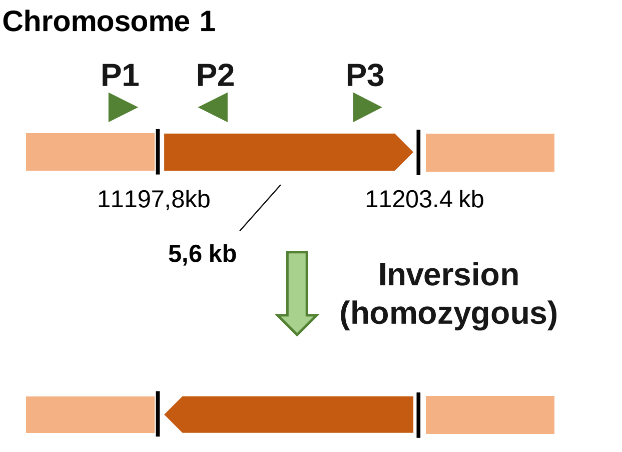

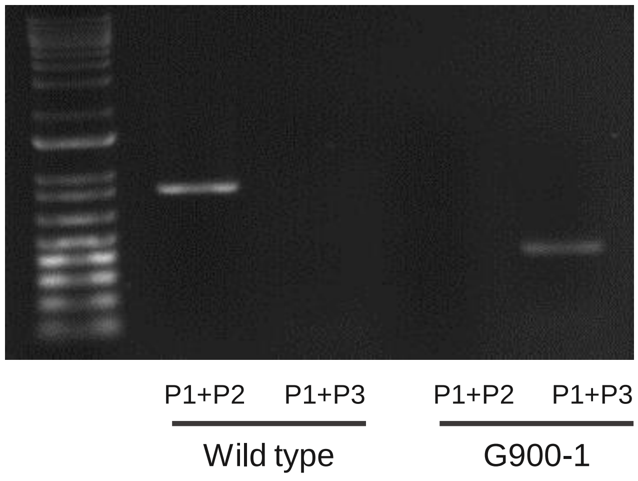

G900-1

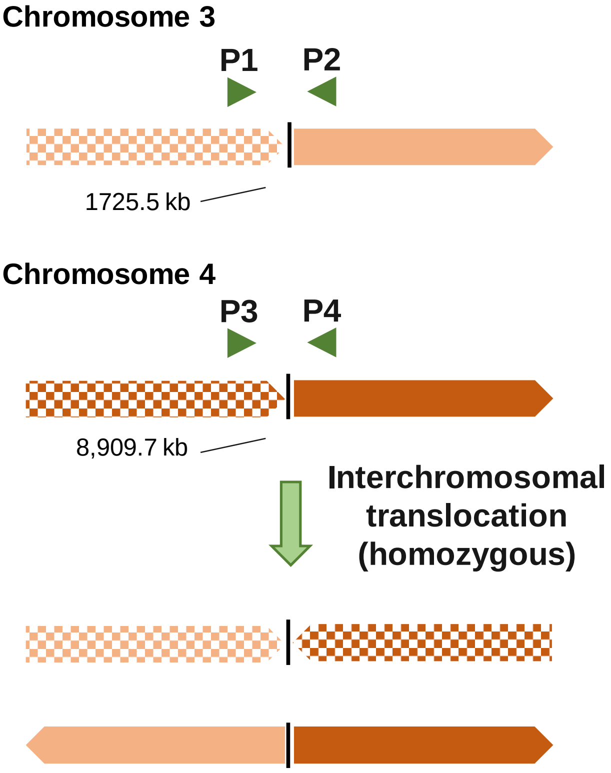

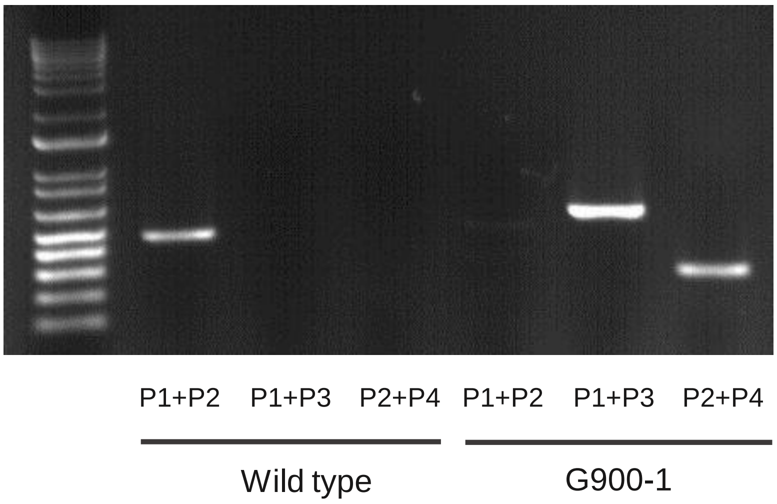

G900-2

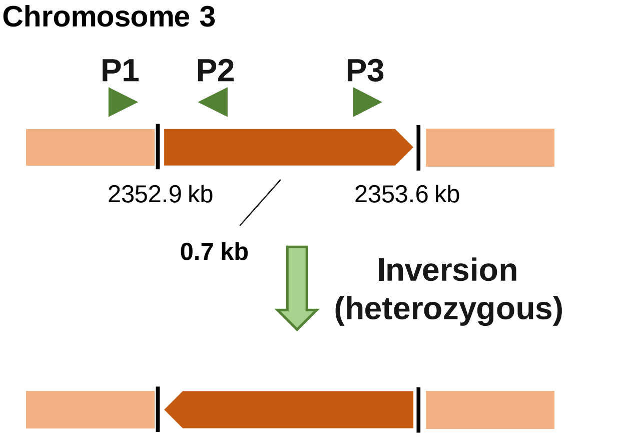

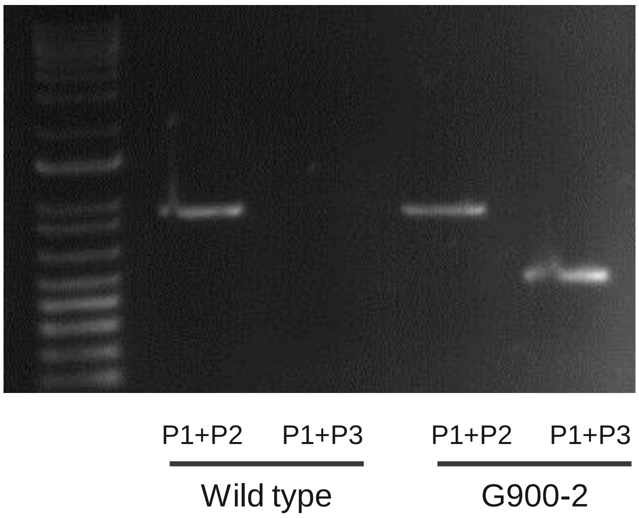

Gamma-rays, 900 Gy (2)

Sample Number	Schematic representation of structural variations	Validation by PCR
G900-5	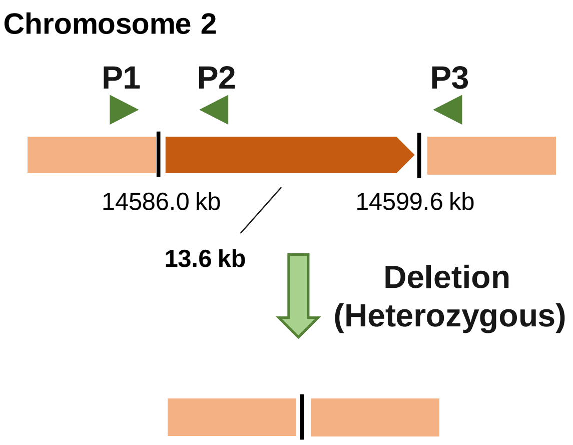	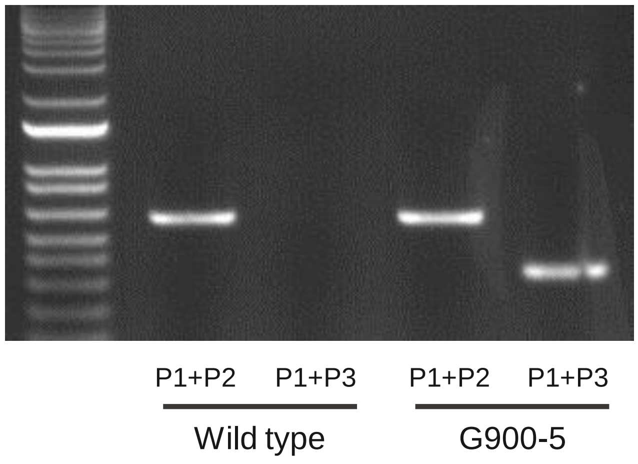
