# Supplementary material for: Mutagenic Effect of Proton Beams Characterized by Phenotypic Analysis and Whole Genome Sequencing in Arabidopsis
Source: Front Plant Sci. 2021 Oct 28;12:752108. doi: 10.3389/fpls.2021.752108 (PMC8581144; doi:10.3389/fpls.2021.752108)

Supplementary Figure S2. Schematic diagram showing sequences around rejoined junctions. Brown and green circles indicate the microhomologous sequences on each rearrangement site in the original molecule. Blue circles in the inversion products indicate nucleotides that may have originated from any microhomologous sequences in the original molecule. The ranges of DNA regions in which DNA breaks or end-joining could occur are indicated by vertical lines. The relative direction of the DNA region is indicated above the DNA sequences. Asterisks indicate nucleotides that are not conserved between microhomologous sequences.

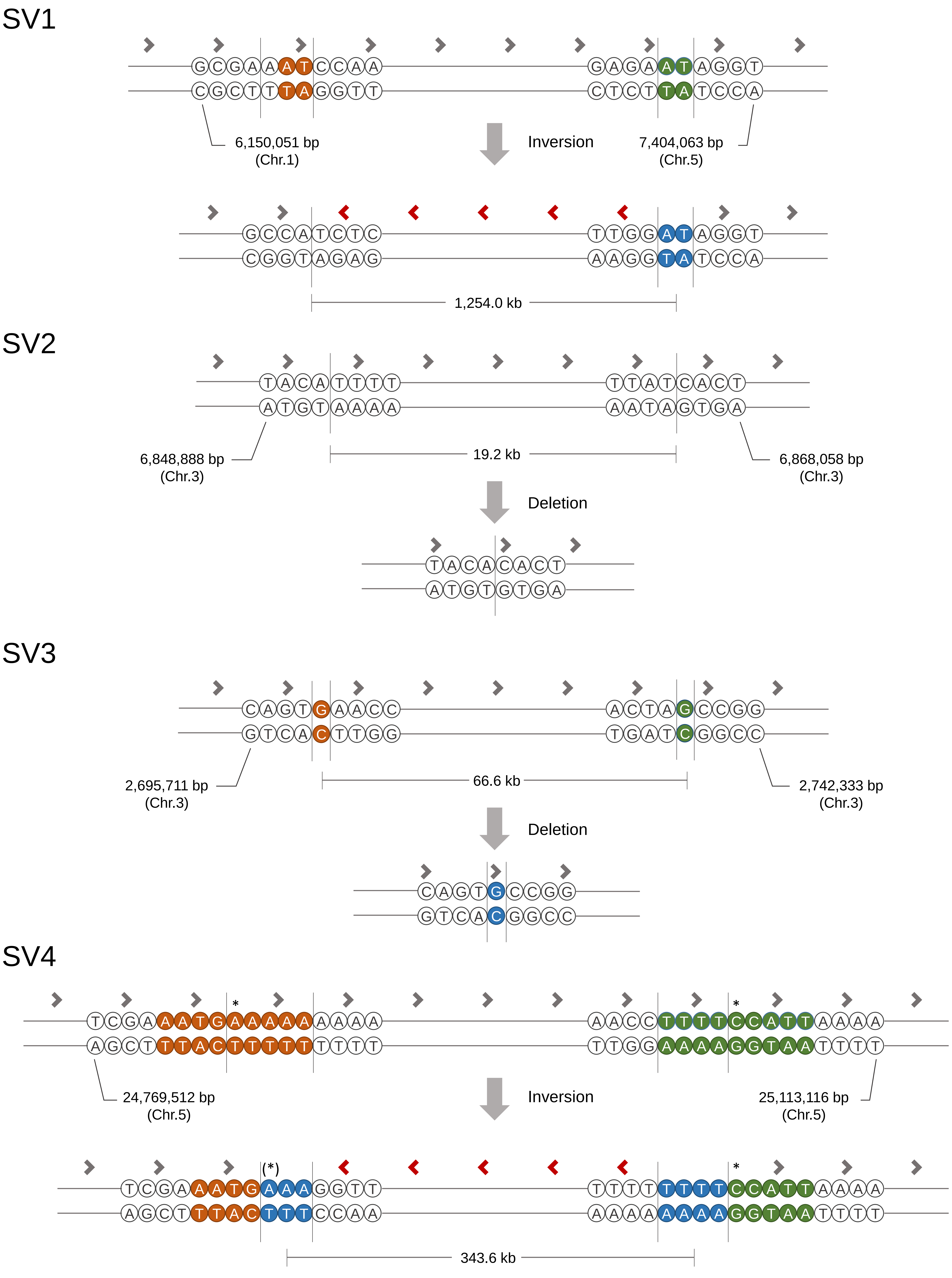

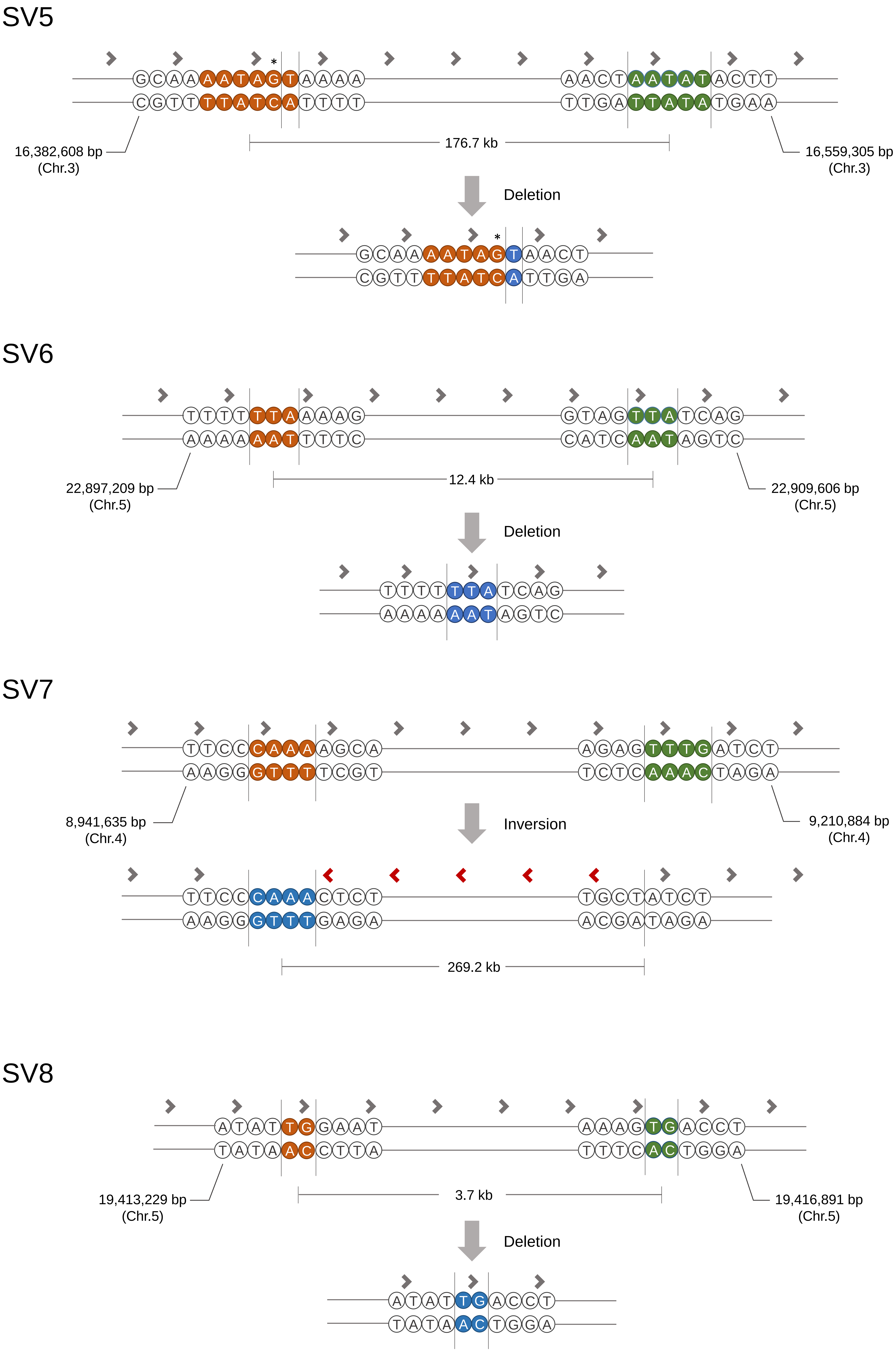
SV5
GCAAAATAGTAAAA
CGTTTTATCATTTT
AACTAATATACTT
TTGATTATATGAA
*
16,382,608 bp
(Chr.3)
176.7 kb
16,559,305 bp
(Chr.3)
Deletion
*
GCAAAATAGTAACT
CGTTTTATCATTGA
SV6
TTTTTTAAAAG
AAAAAATTTTC
GTAGTTATCAG
CATCAATAGTC
22,897,209 bp
(Chr.5)
12.4 kb
22,909,606 bp
(Chr.5)
Deletion
TTTTTTATCAG
AAAAAATAGTC
SV7
TTCCCAAAAGCA
AAGGGTTTTCGT
AGAGTTTGATCT
TCTCAAACTAGA
8,941,635 bp
(Chr.4)
Inversion
9,210,884 bp
(Chr.4)
TTCCCAAACTCT
AAGGGTTTGAGA
TGCTATCT
ACGATAGA
269.2 kb
SV8
ATATTGGAAT
TATAACCTTA
AAAGTGACCT
TTTCACTGGA
19,413,229 bp
(Chr.5)
3.7 kb
19,416,891 bp
(Chr.5)
Deletion
ATATTGACCT
TATAACTGGA

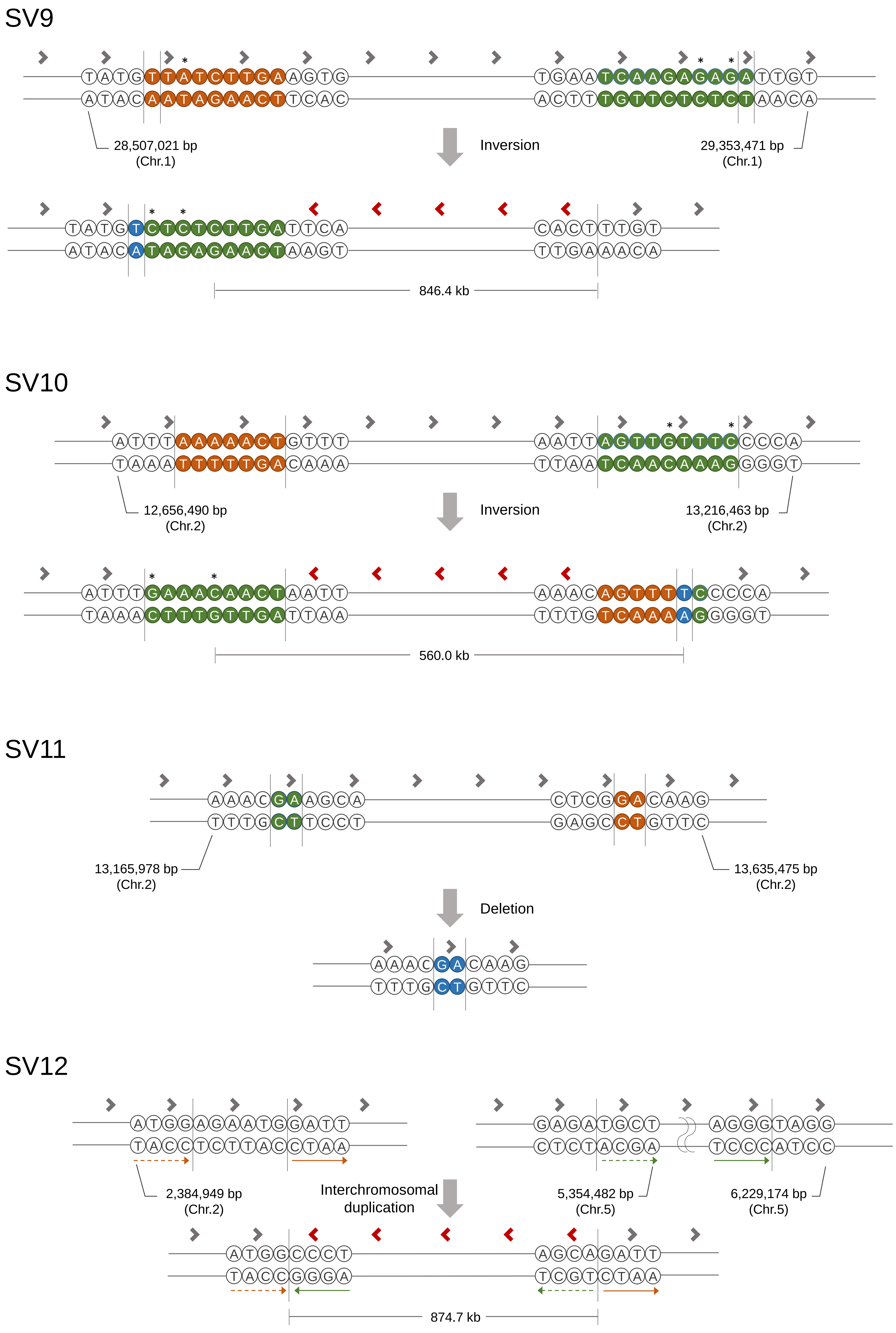
SV9
28,507,021 bp
(Chr.1)
Inversion
29,353,471 bp
(Chr.1)
846.4 kb
SV10
12,656,490 bp
(Chr.2)
Inversion
13,216,463 bp
(Chr.2)
560.0 kb
SV11
13,165,978 bp
(Chr.2)
13,635,475 bp
(Chr.2)
Deletion
SV12
2,384,949 bp
(Chr.2)
Interchromosomal
duplication
5,354,482 bp
(Chr.5)
6,229,174 bp
(Chr.5)
874.7 kb

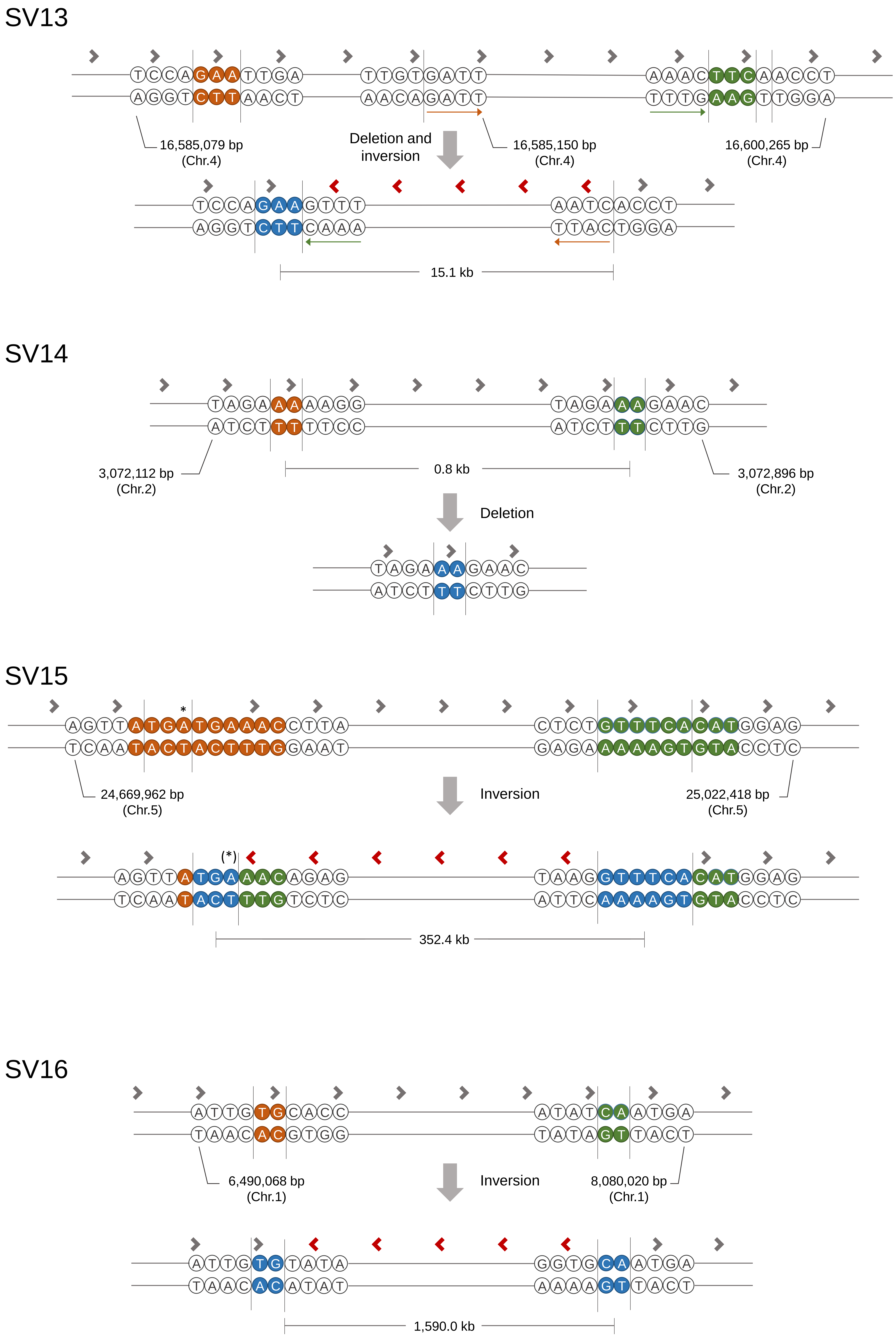
SV13
TCCAGAATTGA
AGGTCTTAACT
TTGTGATT
AACAGATT
AAACTTCAACCT
TTTGAAGTTGGA
16,585,079 bp
(Chr.4)
Deletion and
inversion
16,585,150 bp
(Chr.4)
16,600,265 bp
(Chr.4)
TCCAGAAGTTT
AGGTCTTCAAA
AATCACCT
TTACTGGA
15.1 kb
SV14
TAGAAAAAGG
ATCTTTTTCC
TAGAAAGAAC
ATCTTTCTTG
3,072,112 bp
(Chr.2)
0.8 kb
3,072,896 bp
(Chr.2)
Deletion
TAGAAAGAAC
ATCTTTCTTG
SV15
*
AGTTATGATGAAACCTTA
TCAATACTACTTTGGAAT
CTCTGTTTCACATGGAG
GAGAAAAAGTGTACCTC
24,669,962 bp
(Chr.5)
Inversion
25,022,418 bp
(Chr.5)
(*)
AGTTATGAAACAGAG
TCAATACTTTGTCTC
TAAGGTTTCACATGGAG
ATTCAAAAGTGTACCTC
352.4 kb
SV16
ATTGTGCACC
TAACACGTGG
ATATCAATGA
TATAGTTACT
6,490,068 bp
(Chr.1)
Inversion
8,080,020 bp
(Chr.1)
ATTGTGTATA
TAACACATAT
GGTGCAATGA
AAAAGTTACT
1,590.0 kb

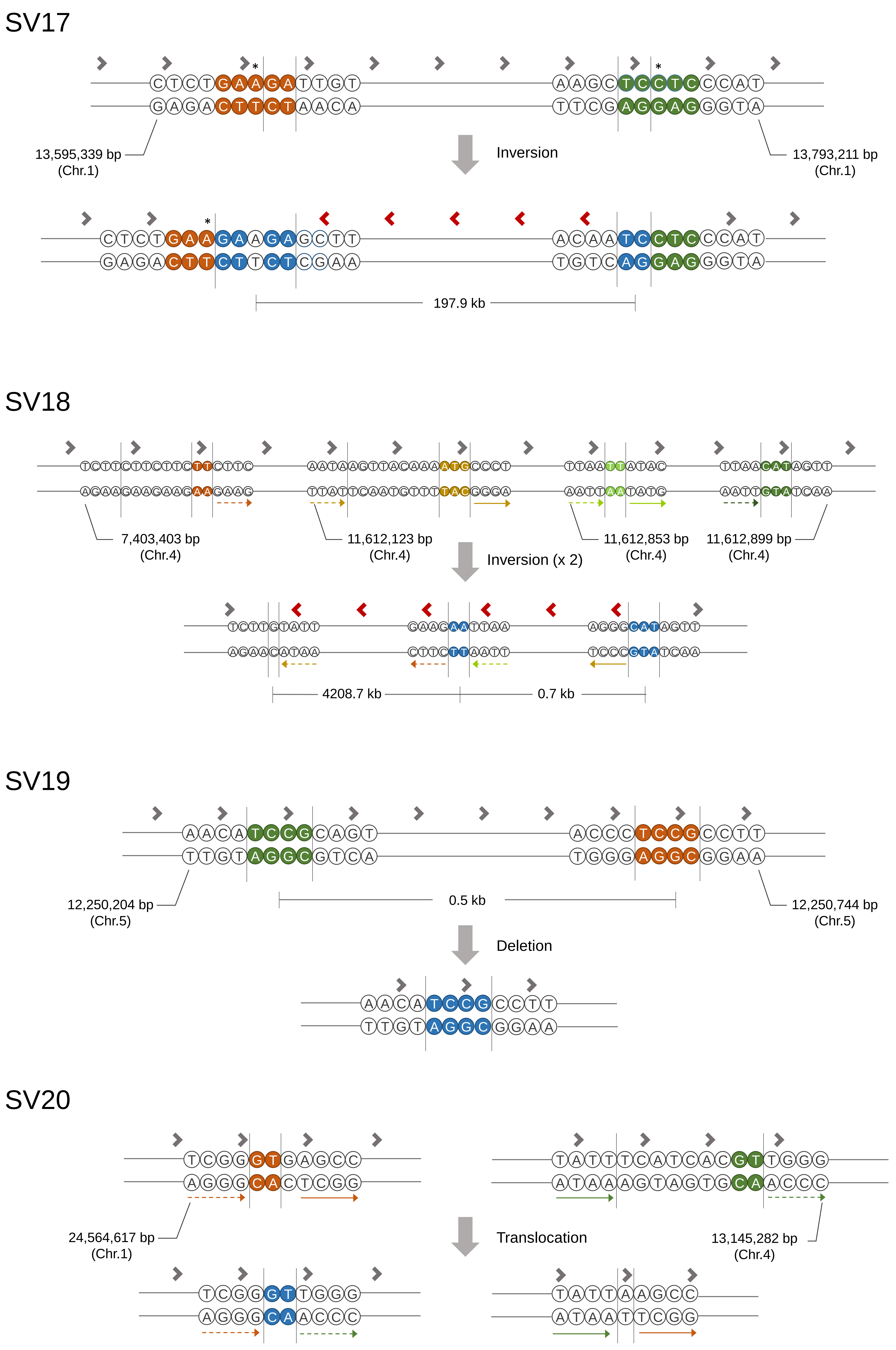
SV17
13,595,339 bp
(Chr.1)
Inversion
13,793,211 bp
(Chr.1)
197.9 kb
SV18
7,403,403 bp
(Chr.4)
11,612,123 bp
(Chr.4)
11,612,853 bp
(Chr.4)
11,612,899 bp
(Chr.4)
Inversion (x 2)
4208.7 kb
0.7 kb
SV19
12,250,204 bp
(Chr.5)
0.5 kb
12,250,744 bp
(Chr.5)
Deletion
SV20
24,564,617 bp
(Chr.1)
Translocation
13,145,282 bp
(Chr.4)

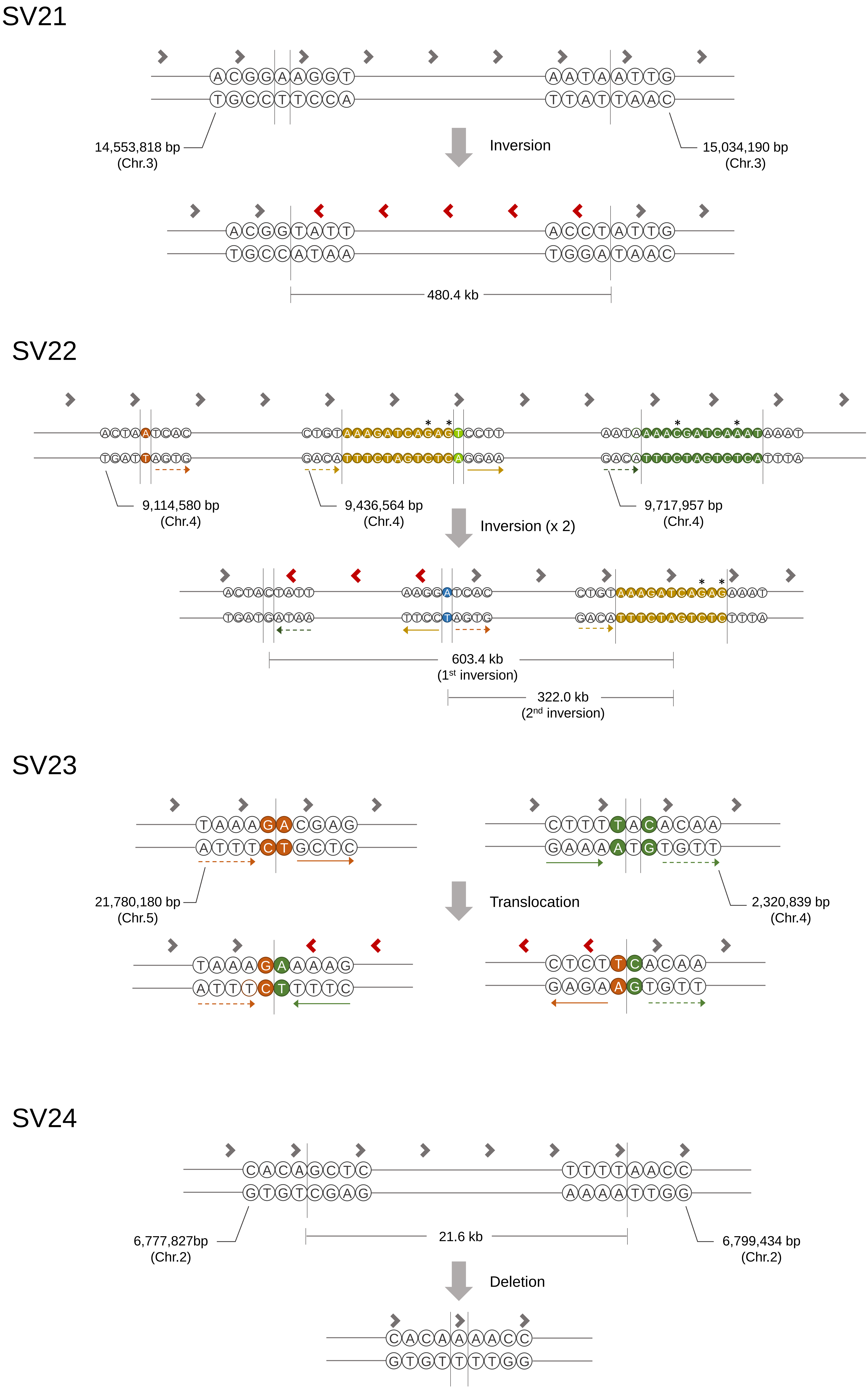
SV21
14,553,818 bp
(Chr.3)
Inversion
15,034,190 bp
(Chr.3)
480.4 kb
SV22
9,114,580 bp
(Chr.4)
9,436,564 bp
(Chr.4)
9,717,957 bp
(Chr.4)
Inversion (x 2)
603.4 kb
(1st inversion)
322.0 kb
(2nd inversion)
SV23
21,780,180 bp
(Chr.5)
Translocation
2,320,839 bp
(Chr.4)
SV24
6,777,827bp
(Chr.2)
21.6 kb
6,799,434 bp
(Chr.2)
Deletion

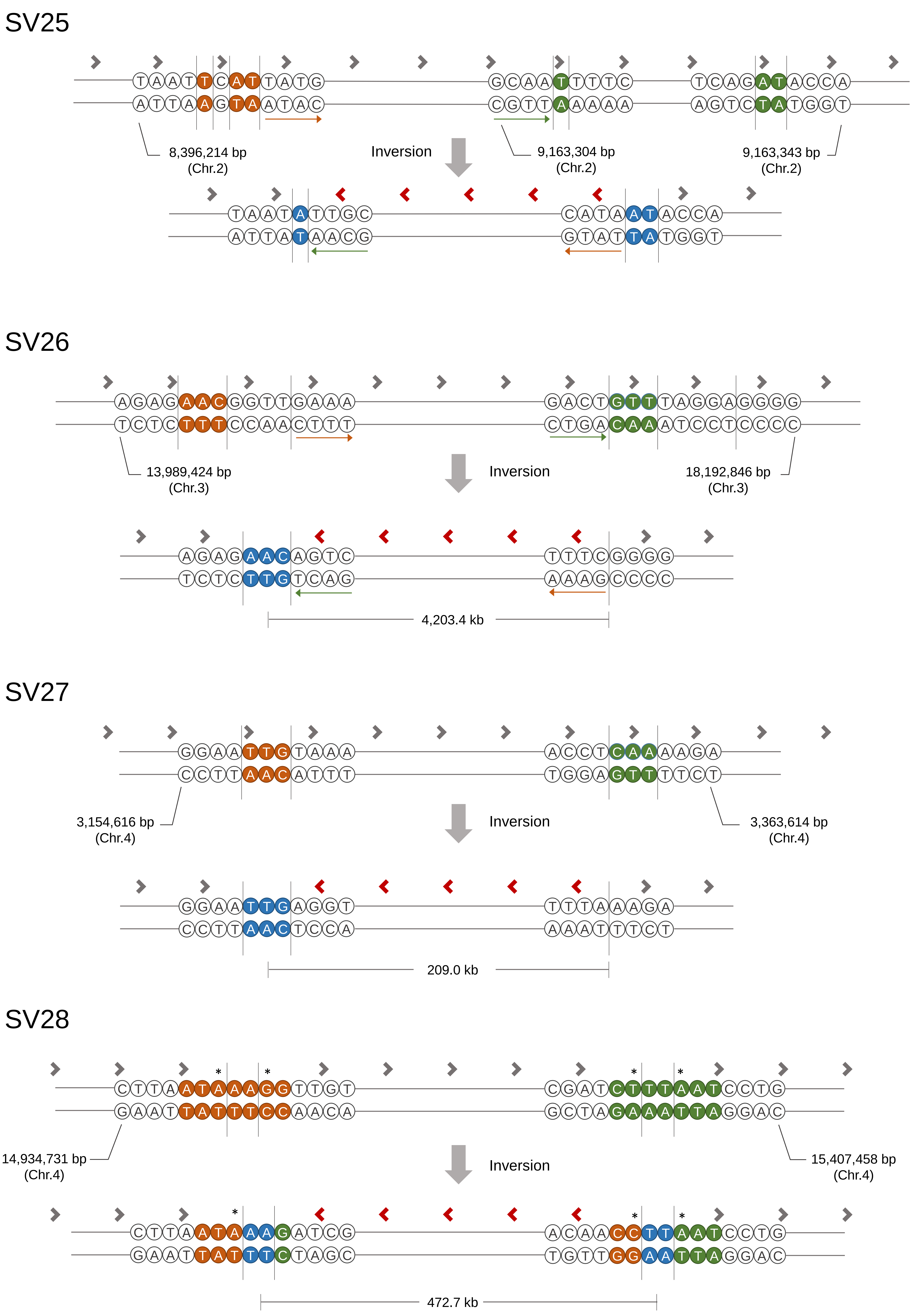

SV25
TAATTCATTATG
ATTAAGTAATAC
GCAATTTTC
CGTTAAAAA
TCAGATACCA
AGTCTATGGT
8,396,214 bp (Chr.2)
Inversion
9,163,304 bp (Chr.2)
9,163,343 bp (Chr.2)
TAATATTGC
ATTATAACG
CATAATACCA
GTATTATGGT
SV26
AGAGAACGGTTGAAA
TCTCTTTCCAACTTT
GACTGTTTAGGAGGGG
CTGACAAATCCTCCCC
13,989,424 bp (Chr.3)
Inversion
18,192,846 bp (Chr.3)
AGAGAACAGTC
TCTCTTGTCAG
TTTCGGGG
AAAGCCCC
4,203.4 kb
SV27
GGAATTGTAAA
CCTTAACATTT
ACCTCAAAAGA
TGGAGTTTTCT
3,154,616 bp (Chr.4)
Inversion
3,363,614 bp (Chr.4)
GGAATTGAGGT
CCTTAACTCCA
TTTAAAGA
AAATTTCT
209.0 kb
SV28
CTTAATAAAGGTTGT
GAATTATTTCCAACA
CGATCTTTAATCCTG
GCTAGAAATTAGGAC
14,934,731 bp (Chr.4)
Inversion
15,407,458 bp (Chr.4)
CTTAATAAAGATCG
GAATTATTTCTAGC
ACAACCTTAATCCTG
TGTTGGAATTAGGAC
472.7 kb

SV29

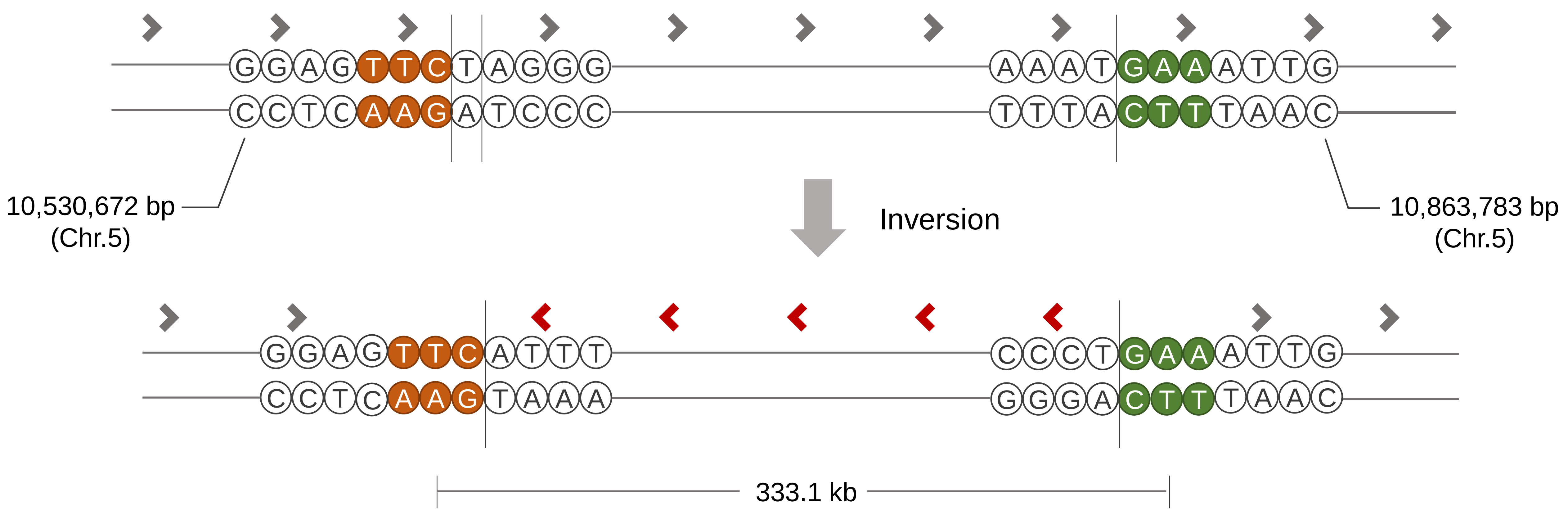

SV30

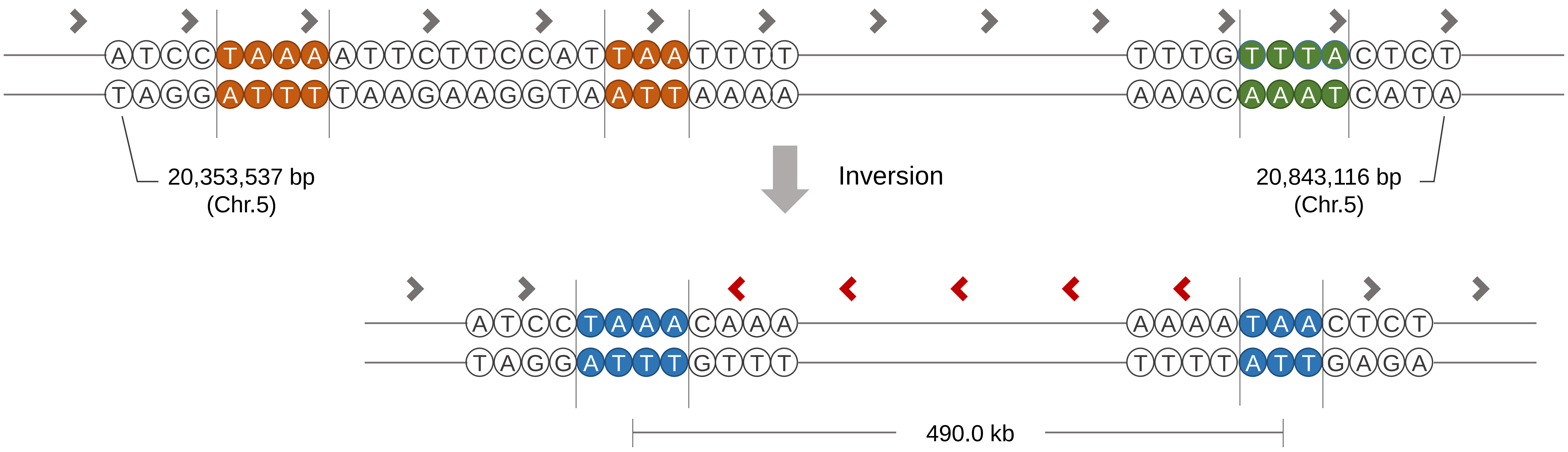

SV31

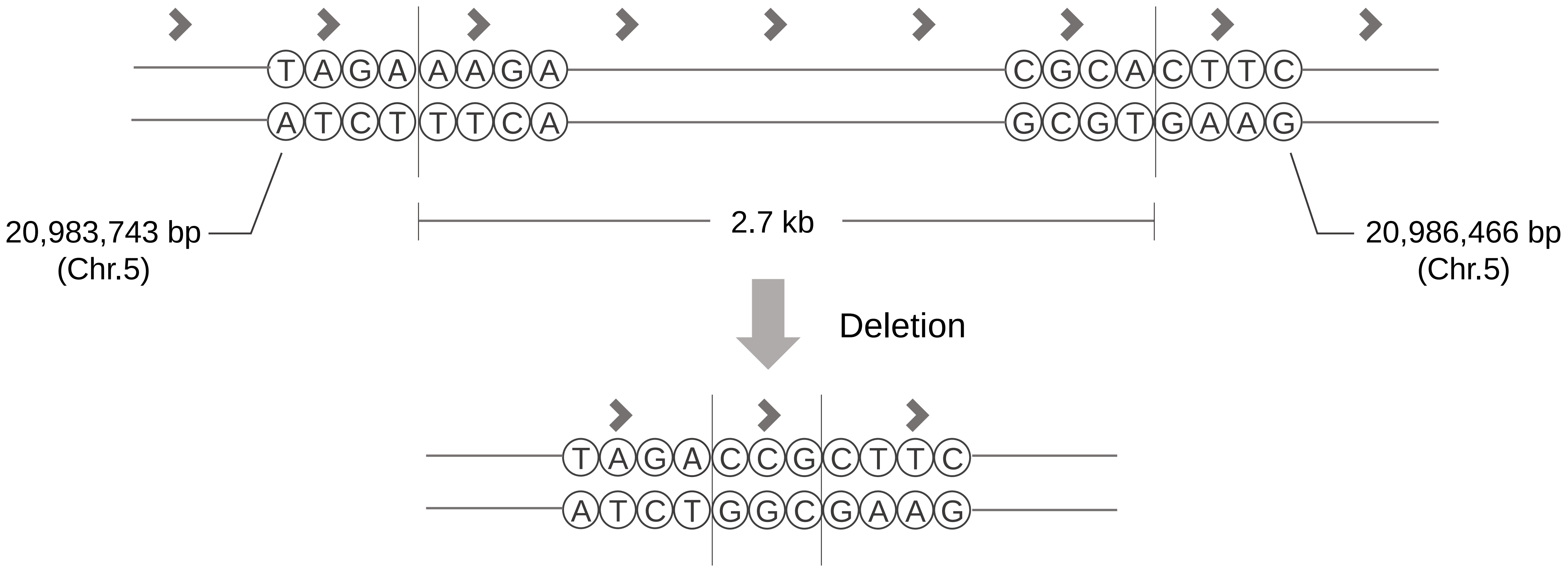

SV32

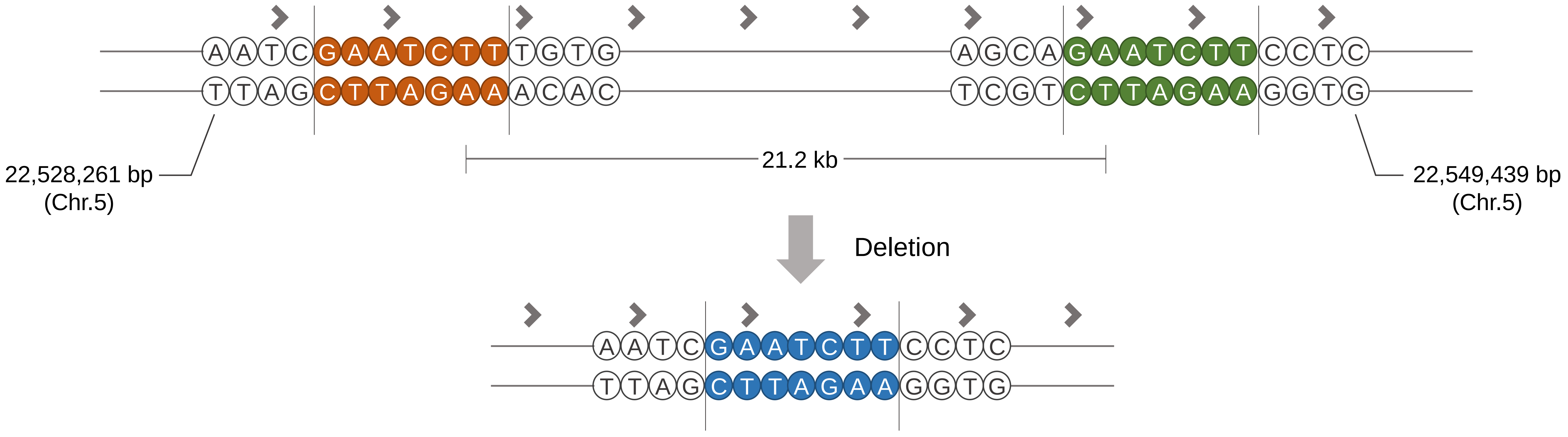

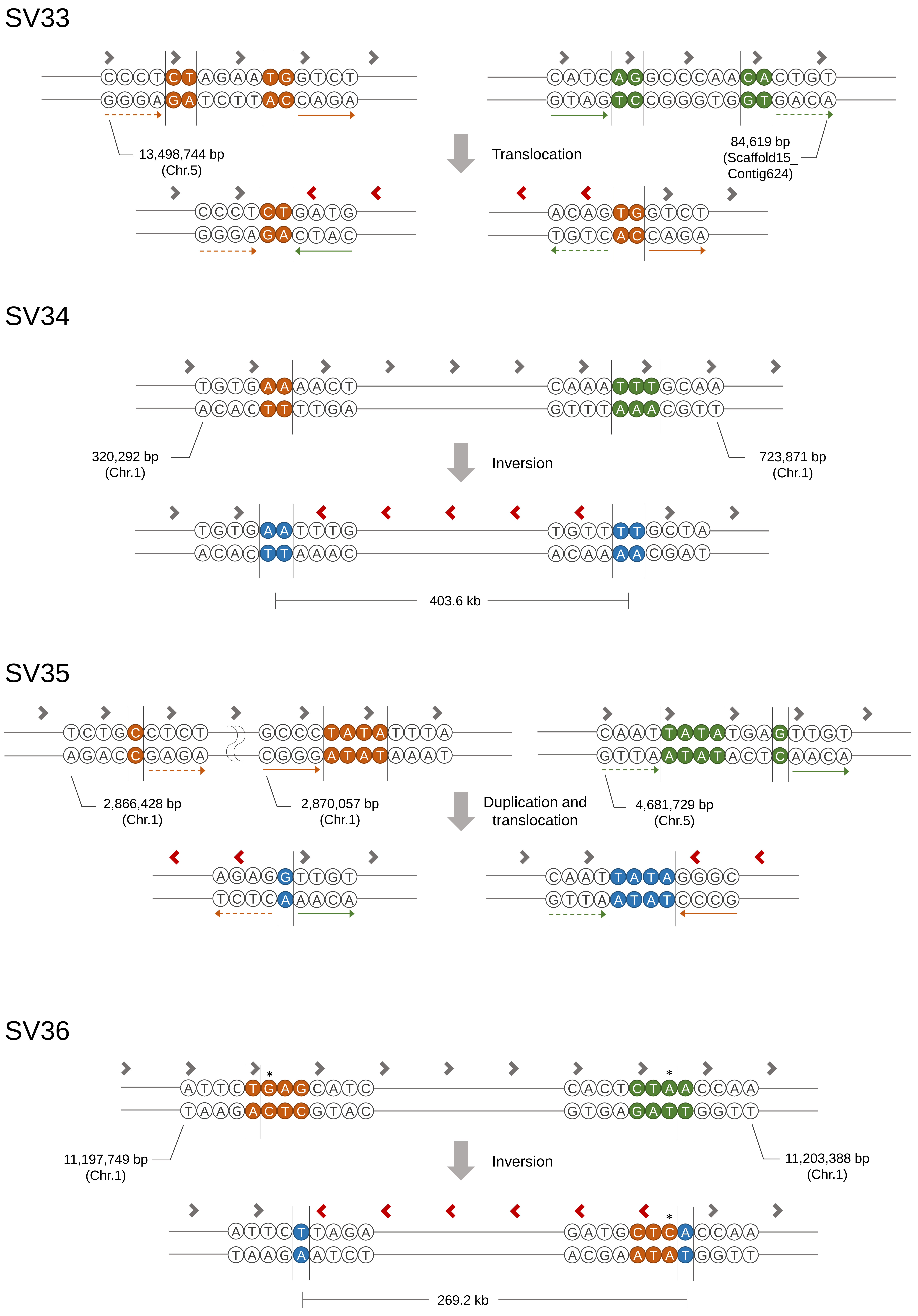
SV33
13,498,744 bp
(Chr.5)
Translocation
84,619 bp
(Scaffold15_
Contig624)
SV34
320,292 bp
(Chr.1)
Inversion
723,871 bp
(Chr.1)
403.6 kb
SV35
2,866,428 bp
(Chr.1)
2,870,057 bp
(Chr.1)
Duplication and
translocation
4,681,729 bp
(Chr.5)
SV36
11,197,749 bp
(Chr.1)
Inversion
11,203,388 bp
(Chr.1)
269.2 kb

SV37

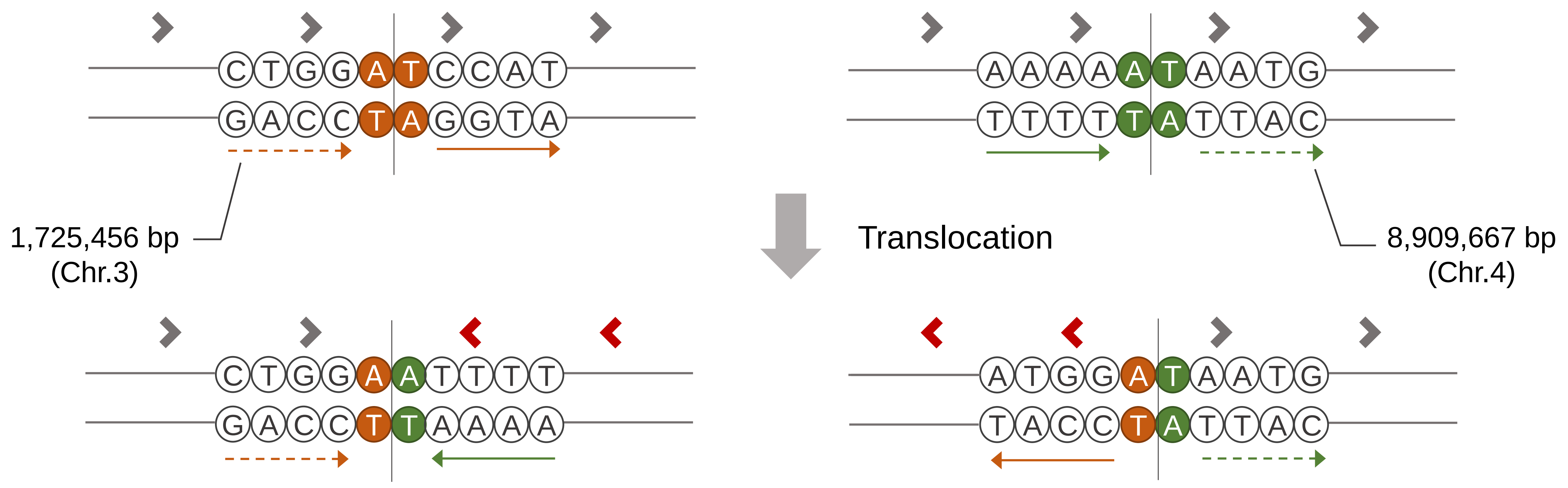

SV38

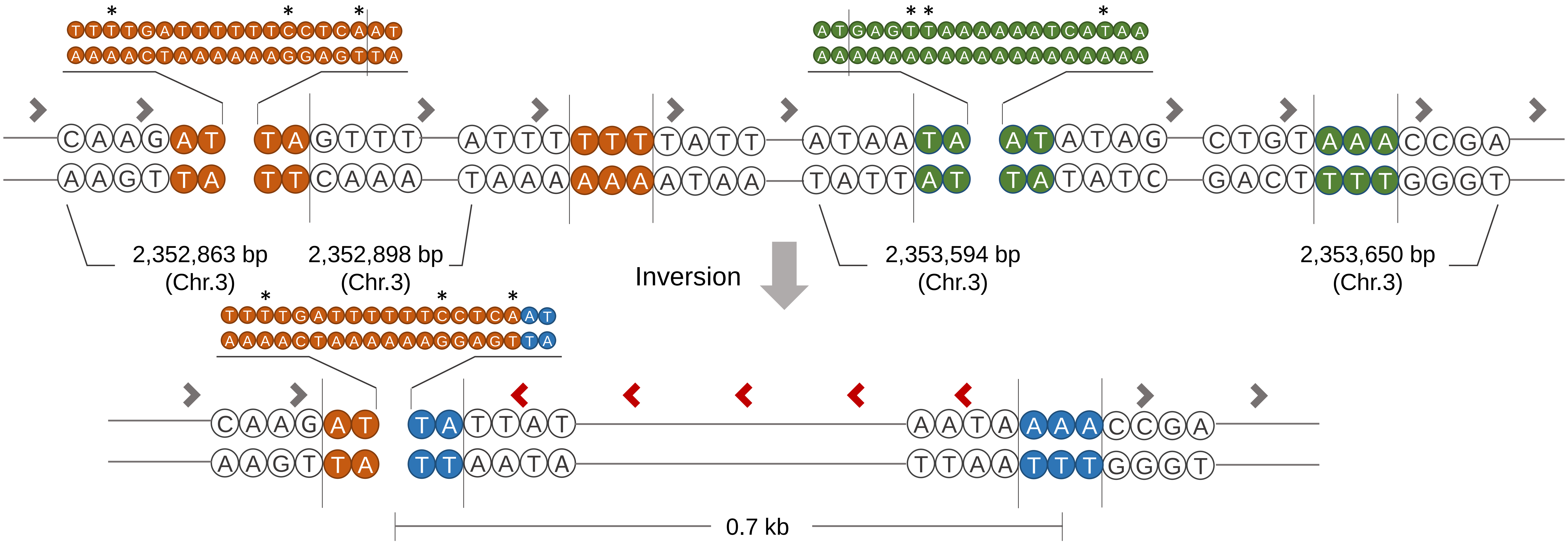

SV39

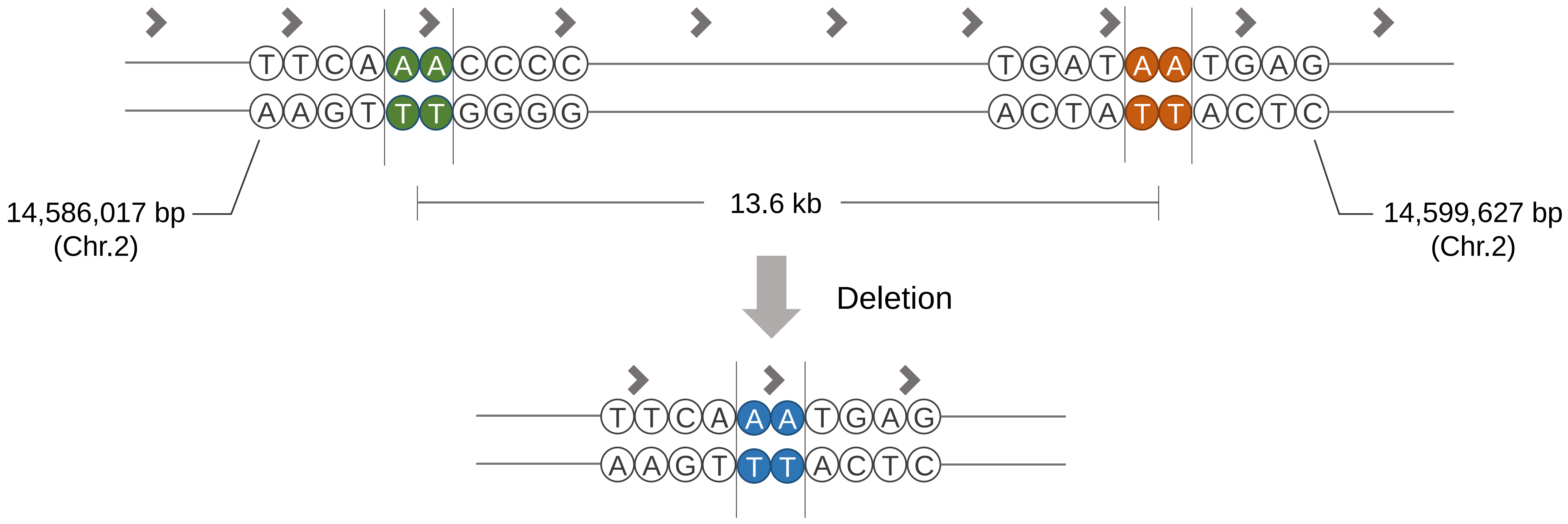

Supplement: Supplementary file 5 [file Data_Sheet_2.PDF]
